# Brazilian fleas (Hexapoda: Siphonaptera): diversity, host associations, and new records on small mammals from the Atlantic Rainforest, including *Rickettsia* screening

**DOI:** 10.1186/s13071-025-06755-6

**Published:** 2025-04-04

**Authors:** Isabella Pereira Pesenato, Jaciara de Oliveira Jorge Costa, Fernando de Castro Jacinavicius, Ricardo Bassini-Silva, Herbert Sousa Soares, Thiago Fakelmann, Giovanna Nosberto Castelli, Gledson Bandeira Maia, Valeria Castilho Onofrio, Fernanda Aparecida Nieri-Bastos, Arlei Marcili

**Affiliations:** 1 Departamento de Biologia Animal, Instituto de Biologia, Universidade Estadual de Campinas, Campinas, SP Brazil; 2 Departamento de Medicina Veterinária Preventiva e Saúde Animal, Faculdade de Medicina Veterinária e Zootecnia, Universidade de São Paulo, São Paulo, SP Brazil; 3 Laboratório de Coleções Zoológicas, Instituto Butantan, São Paulo, SP Brazil; 4 Programa de Mestrado e Doutorado em Saúde Única, Universidade Santo Amaro, São Paulo, SP Brazil; 5 Laboratório de Entomologia em Saúde Pública, Faculdade de Saúde Pública, Universidade de São Paulo, São Paulo, SP Brazil; 6 Faculdade Anclivepa, Medicina Veterinária, São Paulo, SP Brazil

**Keywords:** Ectoparasite, *Rickettsia*, Vector, Brazil

## Abstract

**Background:**

Insects belonging to the Siphonaptera order are obligatory ectoparasites of vertebrates, including humans. Their life cycle is marked by holometabolous development, and adults are adapted to have a bloodmeal out of their hosts. The objective of this study is to review the families occurring in Brazil with their species and report new records from fleas collected in an Atlantic Rainforest preserved area, including *Rickettsia* sp. monitoring.

**Methods:**

Literature research was carried out, including journal articles and books available in scientific databases. The sample collection took place at Legado das Águas—Reserva Votorantim private reserve, where wild rodents, marsupials, and bats were captured and inspected for the presence of fleas. The fleas were identified, and their genetic material was extracted and subjected to two polymerase chain reactions (PCRs): an endogenous control to validate the extraction and a *Rickettsia* screening.

**Results:**

A total of 8 families were reviewed, resulting in 63 valid species that interact with a wide range of hosts. Among the collected fleas, 7 species were identified as interacting with 19 different host genera belonging to the Rodentia, Didelphimorphia, and Chiroptera orders. We highlight the presence of 2 new locality records and 15 new host interactions. Of the collected fleas, 105 specimens were tested individually for *Rickettsia* bacteria, but none showed expected amplicons for the bacterium.

**Conclusions:**

This study provides an extensive revision of the Siphonaptera order present in Brazil with new insights, since the last robust revision made was from 2000, along with new information regarding host association and locality based on field collections conducted by the authors, which helps understanding the host-parasite interaction and encourages new studies.

**Graphical Abstract:**

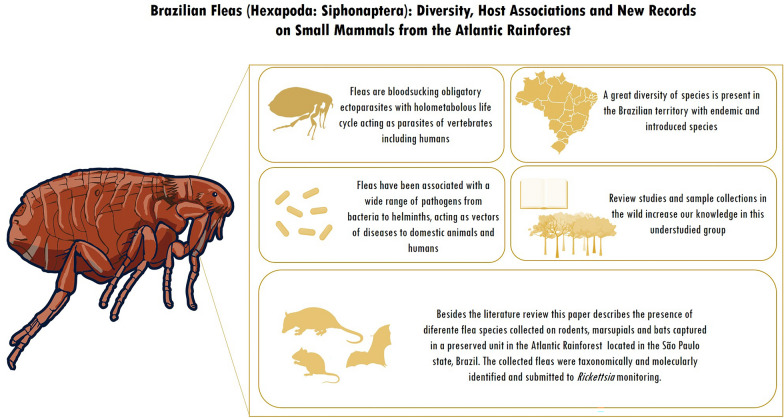

**Supplementary Information:**

The online version contains supplementary material available at 10.1186/s13071-025-06755-6.

## Background

The order Siphonaptera Latreille 1825 belongs to the class Insecta, with three pairs of legs and a body divided into a head, thorax, and abdomen. This order’s main difference is that they have flattened laterally, their hind legs have adapted for jumping, and they lack wings. These are small insects, typically brown in color, that may or may not possess ctenidia, which are specialized structures that facilitate attachment to the host [1].

Fleas are holometabolous, and under ideal conditions, the cycle is completed in approximately 30 days. The stages are egg, larva (with three instars), pupa, and adult. Sexual dimorphism between males and females is present, with females being larger than males. In the adult stage, the mouthparts are sucking-pungitive, performing a solenophagous blood meal on their hosts [2]. Adults are usually found on hosts or in their nests, especially after they have fed, as females need this factor for ovarian maturation and oviposition [3]. While the larvae are legless and vermiform, they have well-developed chewing-type mouthparts due to the basis of their diet being organic matter, including adult feces [4].

Fleas parasitize mainly mammals associated mostly with rodents, bats, and marsupials, with a minority associated with birds [5]. The parasite‒host relationship can be classified as specific (when associated with one order of vertebrates) or generalist (when associated with different hosts orders). Fleas considered generalists are in the minority [1], however, they constitute the greatest concern in public health because they exchange hosts and may be able to transmit pathogens to humans or animals [6, 7].

Fleas have a worldwide distribution, including Antarctica, where *Glaciopsyllus antarticus* Smit and Dunnet 1962 (Ceratophyllidae) was described parasitizing birds [8]. A greater diversity of species has been observed in temperate regions [9] and it is believed that this group has existed since the Eocene, after some researchers found evidence of fossils of mammals from this period [10].

With respect to their permanence on the host, fleas can be subdivided into three categories. (1) Penetrating fleas introduce the head, thorax, and part of the abdomen into the epidermis of the host, forming a neosome around it as it feeds and is filled with blood. After entirely feeding, it lays eggs in the environment and finally dies inside the host. An example of this habit is the species *Tunga penetrans* Linnaeus, 1758 (Tungidae) [11]. (2) The fleas of the genus *Ctenocephalides* Stiles and Collins, 1930 (Pulicidae: Archaeopsyllinae) live most of the time on their hosts, even while not feeding [12]. (3) Fleas that climb on their hosts only to feed but spend most of their adult stage living in mammalian nests, as observed in the genus *Pulex* Linnaeus, 1758 (Pulicidae: Pulicinae) [13].

These ectoparasites can cause several disorders in their hosts, causing itching, discomfort, and dermatitis, spoliative actions that are observed in large infestations that can lead the host to profound anemia and death, in addition to the inflammatory actions observed in infestations by penetrating fleas or semipenetrating events that act as a gateway for opportunistic pathogens, such as bacteria present on the surface of the skin [14]. In addition, fleas are of great medical importance because they act as vectors or intermediate hosts of a wide range of pathogens, such as viruses, bacteria, protozoa, and helminths, that can cause death in animals and humans [15, 16].

Currently, more than 3000 known species are grouped into 240 genera distributed throughout the world [7]. In Brazil, there are approximately 63 species, of which 49 are found to mainly interact with rodents and marsupials, and in addition to generalist fleas, the Ischnopsyllidae family represents the one with highest specificity levels when it comes to host, interacting only with vertebrates of the Chiroptera order. We reviewed the eight leading families, including the endemic species, through a detailed bibliographic review and additionally contributed with new records of fleas collected from small mammals from a private reserve located within the Atlantic Rainforest in southeastern Brazil.

## Methods

### Review

This review was carried out from April 2022 to October 2024 and is based on the work carried out by Lewis (1998) and Linardi and Guimarães (2000), who provided extensive and comprehensive literature reviews on the global and Brazilian flea fauna, respectively. Along with these two fundamental references, we carried out a search for articles available in the PubMed and Google Scholar databases, with keywords in Portuguese and English, and often used combined words such as “flea AND Brazil,” “Brazil AND Siphonaptera,” and “pulgas AND Brasil.” Several articles were identified on the basis of the association of these arthropods with the transmission of infectious diseases, e.g., bubonic plague and murine typhus. Most of the references used were based on the work of two great Brazilian researchers, Dr. Pedro Marcos Linardi and Dr. Lindolpho Rocha Guimaraes, whose work was focused mainly on the order Siphonaptera. This study’s nomenclature and taxonomic division follow those of Linardi and Guimarães [2].

### Specimen collection

The fleas examined were collected in the private reserve Legado das Águas—Reserva Votorantim, Miracatu, São Paulo, Brazil, located in a fragment of the Atlantic Forest, with approximately 75% of the total area composed of dense primary ombrophilous forest. Within the perimeter of the reserve, three areas were chosen for capture: Sede (24° 1′ 49.51″ S, 47° 21′ 8.36″ W), Porto Raso (24° 3′ 25.90″ S, 47° 26′ 30.07″ W), and Serraria (24° 9′ 9.63″ S, 47° 32′ 53.49″ W).

Between January 2018 and December 2021, eight campaigns were conducted, lasting an average of 7–12 days, with three campaigns in the Sede area (January, July, and December 2018), three in Porto Raso (July 2019, February 2020, and October 2021), and two campaigns in Serraria (September and December 2022).

The trails chosen were based on the vegetation and tracks of wild animals. In total, 240 traps (Sherman and Tomahawk traps) were used in each campaign, and small terrestrial mammals were captured via bait made with a mixture of sardines, cornmeal, coconut oil, vanilla, and peanut paste.

For the bat captures, mist nets 3.0 × 6.0 m long were used; the nets were placed at sunset and kept open for 4 h during the sampling nights. For some bat species, an active search was performed at shelters, and the animals were carried inside black fabric bags to the field laboratory.

After capture, the animals were anesthetized with ketamine hydrochloride (15–30 mg/kg), and following sample collection and recovery, the rodents, marsupials, and bats were identified via taxonomic keys [18–20] and then returned to the wild at the same site of capture. All fleas were collected using tweezers and stored in a microtube containing absolute ethanol.

### Flea morphological identification

After collection, the samples were sent to the Laboratório de Coleções Zoológicas of the Instituto Butantan (LCZ-IB) for identification. The fleas were clarified with a 10% potassium hydroxide solution and slide-mounted via Hoyer’s medium. From each batch, only one or two flea specimens were selected for the diaphanization method, while the remaining specimens were preserved in absolute ethanol for posterior molecular analyses. We used Linardi and Guimarães [2] for the identification of genera and species.

Images and measurements were taken with a Leica DM4000B microscope and compiled with Leica Application Suite version 2.5.0. All slide-mounted samples were deposited in the Entomological Collection at the Laboratory of Zoological Collections of the Butantan Institute (LCZ-IB) under the accession numbers IBSP-Ent 14641–IBSP-Ent 14965.

### Molecular analysis

A portion of the collected flea specimens was preserved for subsequent molecular analysis, and EpiInfo™ was utilized to determine the required number of specimens for *Rickettsia* testing on the basis of the expected frequency reported in the literature, with a 99% confidence level. Each flea was individualized in a microtube and subjected to DNA extraction via the commercial DNeasy Blood and Tissue Kit (Qiagen®). The protocol suggested by the manufacturer was followed. After DNA extraction, each exoskeleton was recovered from the columns and slide-mounted for identification following the steps described in the section “flea morphological identification.”

From the extracted DNA, conventional PCRs were performed with primers that amplify an endogenous gene of the flea, mitochondrial cytochrome oxidase II (COII), and the primers F-Leu and R-Lys, which amplify a fragment of 612 bp, as described by Whiting [21]. This PCR was performed only to validate the DNA extraction prior to pathogen testing.

Samples positive for the endogenous gene were considered viable and were thus screened for bacteria of the genus *Rickettsia*. Real-time polymerase chain reaction (qPCR) was performed with the primers CS-5 and CS-6 and an internal probe to amplify a 147 bp fragment of the *gltA* gene, which is present in all bacteria of this genus. This reaction was performed following the protocols of Labruna et al. [22] and Guedes et al. [23]. All reactions included positive (DNA extracted from cell cultures infected with *Rickettsia vini*) and negative (ultrapure water type I) controls.

## Results and discussion

### Literature review

**Fig. 1:**
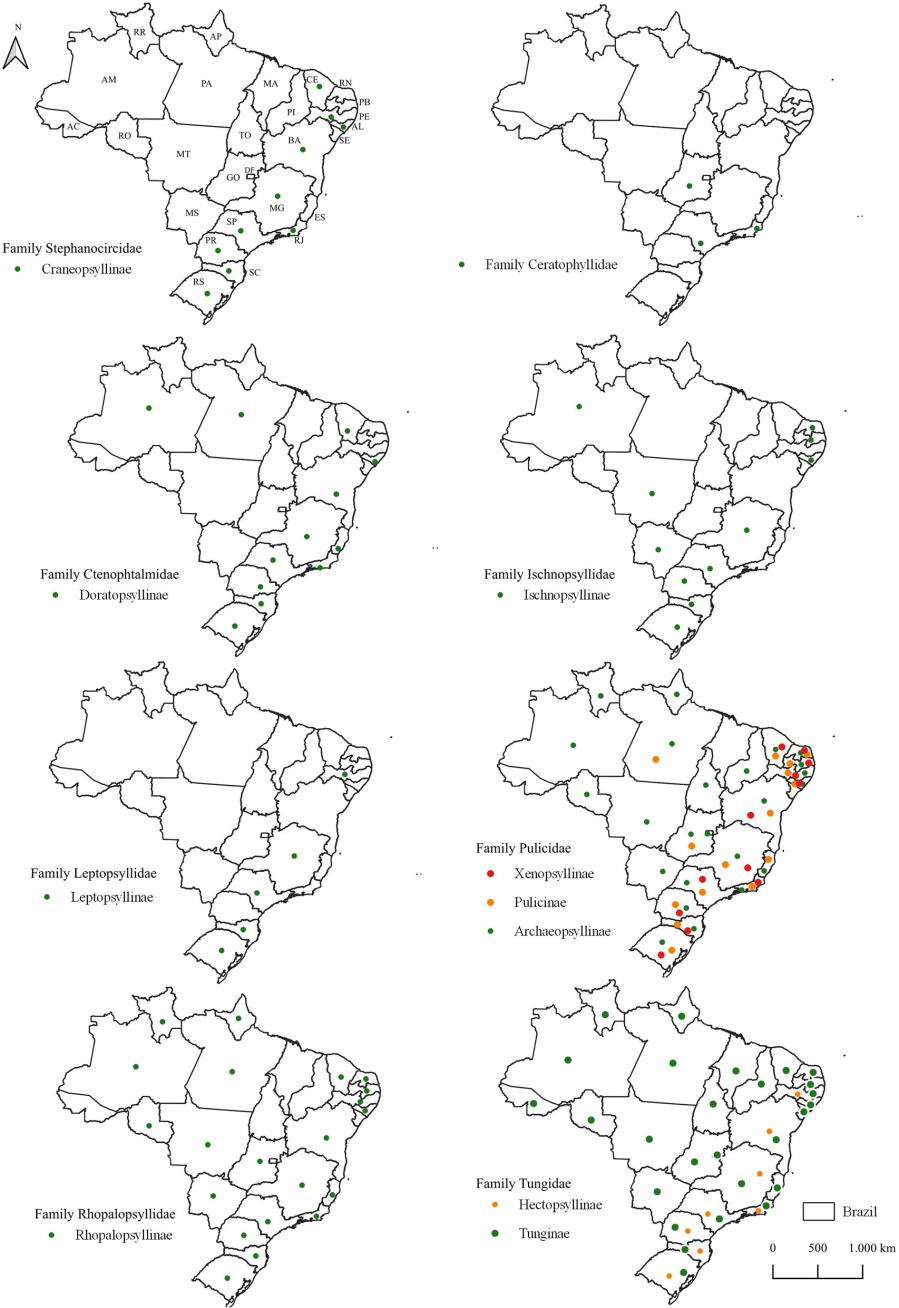
Map showing the distribution of families and subfamilies of fleas in Brazil. States are represented in the map. AC, Acre; AL, Alagoas; AM, Amazonas; AP, Amapá; BA, Bahia, CE, Ceará; DF, Distrito Federal; ES, Espírito Santo; GO, Goiás; MA, Maranhão; MG, Minas Gerais; MS, Mato Grosso do Sul; MT, Mato Grosso; PA, Pará; PB, Paraíba; PE, Pernambuco; PI, Piauí; PR, Paraná; RJ, Rio de Janeiro; RN, Rio Grande do Norte; RO, Rondônia; RS, Rio Grande do Sul; RR, Roraima; SC, Santa Catarina; SE, Sergipe; SP, São Paulo; TO, Tocantins

The data used in this study were extracted from approximately 200 relevant English and Portuguese articles. A total of 8 families of fleas were reported in this review, and these ectoparasites were present in all the 26 states of Brazil. Some species are widely described in the literature; for this reason, the studies were limited by host or locality descriptions, such as the families Pulicidae and Tungidae. This finding is expected, as species within these families exhibit generalist habits regarding both hosts and habitats [17]. Figure 1 represents the localities of families and subfamilies of fleas in the Brazilian states with the literature records and the new records of this study.

### Family Ceratophyllidae Dampf, 1908

This family has 44 genera worldwide, but only the species *Nosopsyllus fasciatus* (Bosc, 1800) occurs in Brazil, and has been associated with synanthropic rodents, such as *Rattus norvegicus* (Berkenhout, 1769) [24]. It can also erratically parasitize dogs and humans, assuming a potential role as a vector of bacterial pathogens [14, 25] as well as protozoan pathogens [26]. This species has been detected with the genetic material of *R. typhi* and could transmit this pathogen among rats, but does not show an important role when it comes to human infection. Concerning plague focus, this species is able to be considered a vector in Southeast Asia and Western Pacific [27]. Currently, this genus is considered cosmopolitan because of human locomotion and the consequent accidental transport of rodents [24].

The health importance of the species *Oropsylla montana* (Baker, 1895) (Ceratophyllinae) stands out. This species has been proven to be able to maintain the bacteria *Yersinia pestis* at low temperatures in North America, which is correlated with a possible role in the epidemiology of plague and Tropical regions [28].

### Family Ctenophtalmidae Rothschild, 1915

The Ctenophtalmidae family is composed of 9 subfamilies, 17 tribes, and 42 genera that include approximately 600 species. Approximately 25% of the flea species described are included in this family. However, new described species that are difficult to classify are also grouped in this family, and because of this, this family may be considered a paraphyletic taxon [9].

The neotropical genus *Adoratopsylla* Ewing 1925 (Doratopsyllinae: Tritopsyllini) is the only one of this family that occurs in Brazil and parasitizes preferably marsupials of the subfamily Didelphinae Gray 1821, but it has already been found in cricetid and sigmodontine rodents [29–32]. This genus is divided into the subgenera *Tritopsylla* Cunha 1914 and *Adoratopsylla*, and five species have been recorded in Brazil: *A. (A.) antiquorum antiquorum* (Rothschild 1904), *A. (A.) antiquorum ronnai* Guimarães 1954, *A. (A.) bisetosa* Ewing 1925, *A. (T.) intermedia intermedia* (Wagner 1901), and *A. (T.) sinuata* Guimarães 1945 [33–35]. Until now, there have been no reports in the literature of fleas of this genus acting as vectors of pathogens of importance in public health or veterinary medicine.

### Family Ischnopsyllidae Tiraboschi, 1904

Ischnopsyllidae are bat ectoparasites associated mainly with the families Vespertilionidae Gray 1821, Molossidae Gervais 1856, and Rhinolophidae Gray 1825, which present very high specificity concerning their hosts, and owing to this association, these fleas have a wide geographic distribution [24, 36]. There is a hypothesis that insectivorous bats would be more susceptible to infestation because they use caves and tree hollows as shelters, which would be more suitable for developing the immature stages of these fleas [37].

The knowledge about the life cycle of the species belonging to this family is still limited, given that the larva has three larval instars and that adults are not known for their ability to jump long distances but for their high climbing ability, mainly because the initial stages of the larva develop through the accumulation of bat feces, and when they leave the pupa, the adults have to climb the walls of the shelters to find the hosts [38–40].

There are 20 genera, 5 tribes, and 2 subfamilies, and these representatives can be found on all continents except Antarctica [24]. Among the 20 known genera, only 5 are described in Brazil; they are distributed among bat species with different eating habits and habitats and are spread across all 5 regions of the country. Table 1 presents these genera, highlighting the species of fleas and hosts and the record status of their occurrence, including our new record of this family.

**Table 1:** Flea species with state locality and hosts described in Brazil, including new records of fleas collected from a preserved Atlantic Rainforest reserve (Legado das Águas—Reserva Votorantim) from 2018 to 2021

Taxon	Host	Brazilian state	References
**Family Ceratophyllidae Dampf, 1908**			
**Genus Nosopsyllus Jordan, 1933**			
*N. fasciatus* (Bosc, 1800)	**Carnivora**: *Canis lupus familiaris* Linnaeus, 1758 **Rodentia**: *Mus musculus* brevirostris (Waterhouse, 1837), *Rattus norvegicus* (Berkenhout, 1769), *Rattus rattus alexandrinus* (Geoffroy, 1803), *Rattus rattus rattus* (Linnaeus, 1758)	GO, RJ, SP	[17]
**Family Ctenophthalmidae Rothschild, 1915**			
**Subfamily Doratopsyllinae Wagner, 1939**			
**Tribe Tritopsyllini Cunha, 1914**			
**Genus *Adoratopsylla* Ewing, 1925**			
**Subgenus *Adoratopsylla* Ewing, 1925**			
*A. (A.) antiquorum antiquorum* (Rothschild, 1904)	**Carnivora**: *Puma yagouaroundi* (Geoffroy, 1803) **Didelphimorphia**: *Didelphis albiventris* Lund, 1840, *Didelphis aurita* (Wied-Neuwied, 1826), *Didelphis marsupialis* Linnaeus, 1758, *Marmosa (Micoureus) paraguayanus* (Tate, 1931), *Marmosa murina* (Linnaeus, 1758), *Marmosops incanus* (Lund, 1840), *Marmosops parvidens* (Tate, 1931), *Metachirus nudicaudatus* (Desmarest, 1817)*, *Monodelphis americana* (Müller, 1776), *Monodelphis dimidiata* (Wagner, 1847), *Monodelphis domestica* (Wagner, 1842), *Monodelphis (Microdelphys) iheringi (*Thomas, 1888), *Philander opossum* (Linnaeus, 1758) **Rodentia:** *Akodon cursor* (Winge, 1887), *Akodon montensis* Thomas, 1913, *Akodon serrensis* Thomas, 1902, *Cerradomys subflavus* (Wagner, 1842), *Delomys dorsalis* (Hensel, 1873), *Delomys sublineatus* (Thomas, 1903), *Euryoryzomys russatus* (Wagner, 1848)*, *Galea spixii* (Wagler, 1831), *Necromys lasiurus* (Lund, 1841), *Nectomys squamipes* (Brants, 1827), *Oligoryzomys moojeni* Weksler & Bonvicino, 2005, *Oligoryzomys nigripes* (Olfers, 1818), *Oxymycterus* sp. Waterhouse, 1837*, *Rhipidomys mastacalis* (Lund, 1840), *Thaptomys nigrita* (Lichtenstein, 1829)*, *Trinomys setosus* (Desmarest, 1817)	AL, BA, CE, ES, MG, PR, RJ, SP*	[17, 30, 33, 35, 106, 110], this study*
*A. (A.) antiquorum ronnai* Guimarães, 1954	**Didelphimorphia:** *Marmosa (Micoureus) paraguayanus*, *Philander opossum*	RS, SC, SP	17
*A. (A.) bisetosa* Ewing, 1925	**Didelphimorphia:** *Monodelphis brevicaudata* (Erxleben, 1777)	AM	17
Subgenus *Tritopsylla* Cunha, 1914			
*A. (T.) intermedia intermedia* (Wagner, 1901)	**Carnivora**: *Cerdocyon thous* (Linnaeus, 1766), *Procyon cancrivorus* Cuvier, 1798 **Didelphimorphia**: Chironectes minimus (Zimmermann, 1780), *Didelphis albiventris*, Didelphis aurita*, *Lutreolina crassicaudata* (Desmarest, 1804), *Marmosa (Marmosa) murina* (Linnaeus, 1758), *Marmosa (Micoureus) paraguayanus, Marmosops incanus*, Metachirus nudicaudatus*, *Monodelphis americana*, *Monodelphis (Microdelphys) iheringi*, *Philander opossum* **Rodentia:** *Cavia aperea* Erxleben, 1777, Euryoryzomys russatus*, *Guerlinguetus* sp. Gray, 1821, *Nectomys squamipes*, *Trinomys paratus* (Moojen, 1948)	BA, ES, MG, PA, PR, RJ, SC, SP*	[17, 30, 33, 35, 69], this study*
*A. (T.) sinuata* Guimarães, 1945	**Didelphimorphia:** Metachirus nudicaudatus*, *Monodelphis* sp. Burnett, 1830*, *Philander opossum* **Rodentia:** ***Euryoryzomys russatus***	PR, SP*	[17], this study*
**Family Ischnopsyllidae Tiraboschi, 1904**			
**Subfamily Ischnopsyllinae Wahlgren, 1907**			
**Tribe Ischnopsyllini Wahlgren, 1907**			
**Genus *Myodopsylla* Jordan & Rothschild, 1911**			
*M. wolffsohni wolffsohni* (Rothschild, 1903)	**Chiroptera**: *Eptesicus* sp. Rafinesque, 1820, *Molossus currentium currentium* Thomas, 1901, *Myotis levis* (Geoffroy, 1824), *Myotis nigricans nigricans* (Schinz, 1821), *Noctilio leporinus* Linneus, 1758	AL, AM, MT, PR, SC	[17, 150]
**Tribe Sternopsyllini Medvedev, 1985**			
**Genus *Hormopsylla* Jordan & Rothschild, 1921**			
*H. fosteri* (Rothschild, 1903)	**Chiroptera**: *Cynomops abrasus abrasus* (Temminck, 1827), *Desmodus rotundus* (Geoffroy, 1810), *Lasiurus (Lasiurus) blossevilli* blossevilli (Lesson, 1826), *Nyctinomops* sp. Miller, 1902*, *Nyctinomops laticaudatus* (Geoffroy, 1805), *Phyllostomus hastatus* Pallas, 1767	MG, PB, RN, SP	[17, 151], this study*
**Genus *Ptilopsylla* Jordan & Rothschild, 1921**			
*P. leptina* Jordan & Rothschild, 1921	**Chiroptera**: *Noctilio albiventris* Desmarest, 1818, *Nyctinomops laticaudatus europs* (Allen, 1889)	MS	[17]
**Genus *Rothschildopsylla* Guimarães, 1953**			
*R. noctilionis* (Costa Lima, 1920)	**Chiroptera**: *Noctilio albiventris*	MS	[17, 103]
**Genus *Sternopsylla* Jordan & Rothschild, 1921**			
*S. distincta distincta* (Jordan & Rothschild, 1921)	**Chiroptera:** *Molossus currentium currentium, Nyctinomops laticaudatus*, *Tadarida brasiliensis* (Geoffroy, 1824)	MG, PR, RS	[17]
**Family Leptopsyllidae Baker, 1904**			
**Subfamily Leptopsyllinae Baker, 1904**			
**Genus *Leptopsylla* Jordan & Rothschild, 1911**			
*L. segnis* (Schönherr, 1811)	**Didelphimorphia**: *Didelphis aurita* **Rodentia**: *Cerradomys subflavus*, *Mus musculus* Linnaeus, 1758, *Oxymycterus delator* Thomas, 1903, *Rattus norvegicus*, *Rattus rattus alexandrinus*, *Rattus* rattus *frugivorus* (Rafinesque, 1814), *Rattus rattus rattus*	MG, PE, RS, SC, SP	[17, 69, 152]
**Family Pulicidae Billberg, 1820**			
**Tribe Archaeopsyllini Oudemans, 1909**			
**Genus *Ctenocephalides* Stiles & Collins, 1930**			
*C. canis* (Curtis, 1826)	**Carnivora**: *Canis lupus familiaris*, *Cerdocyon thous, Felis catus* Linnaeus, 1758	AM, BA, MG, PE, PR, RJ, RS, SC, SP	[17, 105, 108, 111, 153, 154]
*C. felis felis* (Bouché, 1835)	**Artiodactyla**: *Blastocerus dichotomus* Illiger, 1815, *Bos taurus indicus* (Linnaeus, 1758) **Carnivora**: *Canis lúpus familiaris* Linnaeus, 1758, *Cerdocyon thous*, *Chrysocyon brachyurus* (Illiger, 1815), *Eira barbara* (Linnaeus, 1758), *Felis catus*, *Leopardus pardalis* (Linnaeus, 1758), *Leopardus tigrinus* (Schreber, 1775), *Lycalopex vetulus* (Lund, 1842), *Nasua nasua* (Linnaeus, 1766), *Panthera onca* (Linnaeus, 1758), *Procyon cancrivorus*, *Puma yagouaroundi* **Cingulata:** *Dasypus novemcinctus* Linnaeus, 1758 **Didelphimorphia:** *Didelphis albiventris*, *Didelphis aurita*, *Didelphis marsupialis*, *Lutreolina crassicaudata, Marmosa* (*Micoureus*) *paraguayanus*, *Monodelphis domestica* **Lagomorpha:** *Sylvilagus brasiliensis* (Linnaeus, 1758) **Pilosa:** *Tamandua tetradactyla* (Linnaeus, 1758) **Perissodactyla:** *Tapirus terrestris* (Linnaeus, 1758) **Primata:** *Homo sapiens* (Linnaeus, 1758), *Sapajus nigritus* (Goldfuss 1809) **Rodentia:** *Akodon serrensis*, *Cavia porcellus* (Linnaeus, 1758), *Cerradomys subflavus*, *Euryzygomatomys spinosus* (G. Fischer, 1814), *Galea spixii*, *Guerlinguetus aestuans* (Linnaeus, 1766), *Hydrochoerus hydrochaeris* (Linnaeus, 1766), *Necromys* lasiurus, *Oligoryzomys nigripes*, *Oxymycterus dasytrichus* (Schinz, 1821), *Oxymycterus delator*, *Thrichomys laurentius* (Thomas, 1904), *Trinomys albispinus* (Geoffroy, 1838)	AL, AM, AP, BA, CE, ES, DF, GO, MG, MS, MT, PA, PB, PE, PI, PR, RJ, RN, RS, RO, RR, SC, SP, TO	[17, 33, 35, 89, 93, 155, 156, 157, 158, 159, 160, 161]
**Tribe Pulicini Billberg, 1820**			
**Genus *Pulex* Linnaeus, 1758**			
*P. irritans* Linnaeus, 1758	**Carnivora**: *Canis lupus familiaris*, *Cerdocyon thous*, *Conepatus chinga* (Molina, 1782), *Chrysocyon brachyurus*, *Galictis vittata* (Schreber, 1776), *Leopardus geoffroyi* (d'Orbigny & Gervais, 1844), *Leopardus pardalis*, *Panthera onca*, *Procyon cancrivorus* **Chiroptera:** *Nyctinomops laticaudatus* **Pilosa:** *Tamandua tetradactyla* **Primata**: *Homo sapiens* **Didelphimorphia**: *Monodelphis domestica*, *Philander opossum* **Rodentia:** *Cuniculus paca* (Linnaeus, 1766), *Galea spixii*, *Guerlinguetus aestuans*, *Holochilus brasiliensis* (Desmarest, 1819), *Kerodon rupestris* (Wied-Neuwied, 1820), *Oligoryzomys nigripes*, *Thrichomys inermis* (Pictet, 1841), *Thrichomys laurentius*, *Trinomys dimidiatus* (Günther, 1877), *Trinomys setosus*, *Wiedomys pyrrhorhinos* (Wied-Neuwied, 1821)	AL, BA, CE, ES, GO, MG, PA, PB, PE, PI, PR, RJ, RN, RS, SC, SP	[17, 63, 70, 116, 162, 163, 164]
**Tribe Xenopsyllini Glienkiewicz, 1907**			
**Genus *Xenopsylla* Glienkiewicz, 1907**			
*X. brasiliensis* (Baker, 1904)	**Carnivora**: *Canis lupus familiaris* **Rodentia:** *Necromys lasiurus*, *Oligoryzomys nigripes*, *Mus musculus, Rattus norvegicus*, *Rattus rattus alexandrinus*, *Rattus* rattus *frugivorus*, *Rattus rattus rattus*	CE, PB, PE, RN, RJ, RS, SP	[17]
*X. cheopis* (Rothschild, 1903)	**Carnivora**: *Canis lupus familiaris*, *Cerdocyon thous* **Didelphimorphia**: *Didelphis aurita*, *Didelphis marsupialis*, *Monodelphis domestica* **Rodentia**: *Akodon cursor*, *Akodon montensis*, *Cavia aperea*, *Cerradomys subflavus*, *Galea spixii*, *Holochilus brasiliensis*, *Holochilus sciureus* Wagner, 1842, *Necromys lasiurus*, *Nectomys squamipes*, *Rattus rattus rattus*, *Thrichomys apereoides* (Lund, 1839), *Thrichomys inermis*, *Thrichomys laurentius*, *Trinomys elegans* (Lund, 1841), *Trinomys setosus*	AL, BA, CE, MG, PE, PR, RJ, RN, RS, SC, SP	[17, 29, 107, 115, 165, 166, 167]
**Family Rhopalopsyllidae Oudemans, 1909**			
**Subfamily Rhopalopsyllinae Oudemans, 1909**			
**Tribe Polygenini Linardi & Guimarães, 1993**			
**Genus *Neotropsylla* Linardi & Guimarães, 1993**			
*N. guimaraesi* (Linardi, 1978)	**Rodentia**: *Calomys* sp. Waterhouse, 1837	SP	[2, 17]
**Genus *Polygenis* Jordan, 1939**			
**Subgenus *Polygenis* (*Neopolygenis*) Linardi & Guimarães, 1993**			
*P. (N.) atopus* (Jordan & Rothschild, 1922)	**Carnivora**: *Eira barbara, Felis catus, Procyon cancrivorus* **Didelphimorphia:** *Didelphis albiventris*, *Didelphis aurita*, *Didelphis marsupialis*, *Philander opossum* **Passeriformes**: *Haplospiza unicolor* Linnaeus, 1766 **Rodentia**: *Akodon cursor*, *Akodon montensis*, *Caluromys* philander (Linnaeus, 1758), *Cerradomys subflavus*, *Delomys dorsalis*, *Euryoryzomys russatus*, *Guerlinguetus* sp., *Holochilus brasiliensis*, *Nectomys squamipes*, *Oligoryzomys flavescens* (Waterhouse, 1837), *Oligoryzomys nigripes*, *Oxymycterus dasytrichus*, *Rhipidomys mastacalis*, *Sooretamys angouya* (Fischer, 1814)	MG, PR, RS, RJ, SC, SP	[17, 30, 31, 104, 110, 168]
*P. (N.) dentei* Guimarães, 1947	**Rodentia**: *Akodon cursor*, *Akodon montensis*, *Delomys dorsalis*, *Oxymycterus quaestor* Thomas, 1903, *Thaptomys nigrita*	SP, RJ	[2, 17, 169]
*P. (N.) frustratus* Johnson, 1957	**Didelphimorphia**: *Didelphis marsupialis*, *Philander opossum* **Rodentia:** *Akodon montensis*, *Cavia aperea*, *Delomys dorsalis*, *Oxymycterus dasytrichus*, *Oxymycterus quaestor* Thomas, 1903, *Thaptomys nigrita*	SP, RJ, SC, PR	[2, 17, 169]
*P. (N.) pradoi* (Wagner, 1937)	**Carnivora**: *Nasua nasua* **Didelphimorphia**: *Didelphis albiventris*, *Didelphis marsupialis*, *Philander opossum* **Rodentia**: *Akodon cursor*, *Akodon montensis*, *Akodon reigi* González, Langguth & Oliveira, 1998, *Akodon serrensis*, *Euryoryzomys russatus*, *Euryzygomatomys spinosus*, *Necromys lasiurus*, *Nectomys squamipes*, *Oligoryzomys nigripes*, *Oxymycterus quaestor*, *Rattus rattus rattus*, *Thaptomys nigrita*, *Trinomys iheringi* (Thomas, 1911)	BA, ES, PR, RJ, RS, SC, SP	[2, 17, 106, 116, 118, 170]
*P. (N.) pygaerus* (Wagner, 1937)	**Didelphimorphia**: *Didelphis aurita* **Rodentia**: *Akodon cursor*, *Akodon montensis*, *Akodon serrensis*, *Euryoryzomys russatus*, *Necromys lasiurus*, *Nectomys squamipes*, *Oxymycterus quaestor*, *Rattus rattus rattus*, *Thaptomys nigrita*	MG, PR, RJ, SC	[17, 116, 170]
**Subgenus *Polygenis* (*Polygenis*) Jordan, 1939**			
*P. (P.) acodontis* (Jordan & Rothschild, 1923)	**Rodentia**: *Guerlinguetus aestuans*	SC	[17]
*P. (P.) adelus* (Jordan & Rothschild, 1923*)*	**Didelphimorphia**: *Monodelphis domestica* **Rodentia**: *Akodon montensis*, *Calomys tener* (Winge, 1887), *Cerradomys subflavus*, *Euryoryzomys russatus*, *Necromys lasiurus*, *Rhipidomys mastacalis*, *Trinomys albispinus*, *Trinomys setosus*, *Wiedomys pyrrhorhinos*	BA, MG, PE, SP	[2, 17]
*P. (P.) axius axius* (Jordan & Rothschild, 1923)	**Didelphimorphia**: *Didelphis albiventris*, *Lutreolina crassicaudata* **Rodentia**: *Akodon cursor*, *Necromys lasiurus*, *Nectomys squamipes*, *Oxymycterus dasytrichus*	MG, PR, RS, SP	[2, 17, 33]
*P. (P.) axius pessoai* Guimarães, 1956	**Rodentia**: *Cerradomys subflavus*, *Oligoryzomys nigripes*, *Oxymycterus dasytrichus*	AL, PE	[17]
*P. (P.) axius proxima* Guimarães, 1948	**Didelphimorphia:** *Lutreolina crassicaudata* **Rodentia**: *Akodon montensis, Necromys lasiurus*	MG, MS, RS, SP	[2, 17, 103, 168]
*P. (P.) bohlsi bohlsi* (Wagner, 1901)	**Didelphimorphia**: *Didelphis* sp. Linnaeus, 1758 **Rodentia:** *Calomys callosus* (Rengger, 1830), *Calomys tener*, *Cerradomys subflavus*, *Cuniculus paca*, *Necromys lasiurus*, *Nectomys squamipes*, *Oligoryzomys nigripes*, *Oxymycterus dasytrichus*, *Rattus rattus frugivorus*, *Thrichomys apereoides*	ES, GO, MG, MS	[17, 35, 119, 172]
*P. (P.) bohlsi jordani* (Lima, 1937)	**Carnivora**: *Cerdocyon thous*, *Galictis vittata* **Didelphimorphia:** *Didelphis albiventris*, Metachirus nudicaudatus*, *Monodelphis domestica* **Lagomorpha**: *Sylvilagus brasiliensis* **Primata:** *Callithrix jacchus* (Linnaeus, 1758) **Rodentia**: *Akodon montensis*, *Calomys expulsus* (Lund, 1840), *Calomys tener*, *Cavia aperea*, *Cerradomys subflavus*, *Echimys chrysurus* (Zimmermann, 1780), *Euryoryzomys lamia* (Thomas, 1901), Euryoryzomys russatus*, *Galea spixii*, *Holochilus brasiliensis*, *Holochilus sciureus*, *Kerodon rupestris*, *Mus musculus brevirostris*, *Necromys lasiurus*, *Nectomys squamipes*, *Oligoryzomys flavescens*, *Oligoryzomys* sp.*, *Oxymycterus* sp.*, *Rattus norvegicus*, *Rattus rattus alexandrinus*, *Rattus rattus frugivorus*, *Rhipidomys mastacalis*, *Thrichomys inermis*, *Thrichomys laurentius*, *Trinomys albispinus*, *Trinomys setosus*, *Wiedomys pyrrhorhinos*	AL, BA, CE, PE, PB, RN, SP*	[17, 30, 105, 170, 173], this study*
*P. (P.) occidentalis occidentalis* (Jordan & Rothschild, 1923)	**Carnivora**: *Cerdocyon thous* **Cingulata:** *Dasypus novemcinctus* **Didelphimorphia**: *Didelphis aurita*, *Didelphis marsupialis* **Rodentia**: *Akodon* spp. Meyen, 1833, *Delomys dorsalis*, *Guerlinguetus aestuans*, *Guerlinguetus brasiliensis ingrami* (Thomas, 1901), *Necromys lasiurus*, *Oligoryzomys nigripes*, *Oxymycterus nasutus* (Waterhouse, 1837), *Rattus norvegicus*, *Rhipidomys mastacalis*, *Scapteromys tumidus* (Waterhouse, 1837), *Thrichomys inermis* **Tinamiformes:** *Crypturellus obsoletus obsoletus* (Sclater, 1865)	AL, CE, ES, PR, RJ, RS, SC, SP	[2, 17, 30, 35, 118]
*P. (P.) occidentalis steganus* (Jordan & Rothschild, 1923)	**Rodentia:** *Rhipidomys mastacalis*	CE, GO, PA, RR	[17]
*P. (P.) platensis platensis* (Jordan & Rothschild, 1908)	**Rodentia**: *Akodon azarae* (Fischer, 1829), *Ctenomys flamarioni* Travi, 1981, *Ctenomys minutus* Nehring, 1887, *Delomys dorsalis*, *Oligoryzomys nigripes*, *Scapteromys tumidus*	RS	[17, 118, 174]
*P. (P.) rimatus* (Jordan, 1932)	**Didelphimorphia:** *Didelphis albiventris*, *Didelphis marsupialis*, *Monodelphis brevicaudata*, *Philander* sp. Brisson, 1762, Metachirus nudicaudatus* **Rodentia**: *Akodon* sp.*, *Akodon cursor*, *Akodon montensis*, *Akodon serrensis*, *Calomys expulsus*, *Cerradomys subflavus*, *Delomys dorsalis*, Euryoryzomys russatus*, *Euryzygomatomys spinosus*, *Guerlinguetus brasiliensis ingrami*, *Necromys lasiurus*, *Nectomys squamipes*, *Oligoryzomys mattogrossae* Allen, 1916, *Oligoryzomys nigripes*, *Oxymycterus* sp.*, *Oxymycterus quaestor*, *Rattus norvegicus*, *Rattus rattus alexandrinus*, *Sooretamys angouya*, *Thaptomys nigrita*, *Trinomys dimidiatus*	BA, ES, GO, MG, PA, PR, RJ, RS, SC, SP	[17, 30, 33, 104, 106, 110, 116, 118, 168, 170], this study*
*P. (P.) roberti beebei* (Fox, 1947)	**Rodentia:** *Euryoryzomys* spp.	AP	[17]
*P. (P.) roberti roberti* (Rothschild, 1905)	**Carnivora**: *Leopardus pardalis* **Chiroptera**: ***Chrotopterus auritus*** (Peters, 1856)* **Cingulata:** *Dasypus novemcinctus* **Didelphimorphia**: *Didelphis albiventris*, Didelphis aurita*, *Didelphis marsupialis*, *Gracilinanus* sp. Gardner & Creighton, 1989*, *Marmosa* (*Micoureus*) *paraguayanus*, Metachirus nudicaudatus*, *Monodelphis* sp.* **Pilosa:** *Tamandua tetradactyla* **Rodentia**: *Akodon* sp.*, *Akodon montensis*, *Brucepattersonius* sp. Hershkovitz, 1998*, *Cerradomys subflavus*, *Dasyprocta azarae* Lichtenstein, 1823, *Dasyprocta* (Linnaeus, 1758), *Delomys dorsalis*, *Euryoryzomys lamia*, Euryoryzomys russatus*, Guerlinguetus brasiliensis ingrami*, Holochilus brasiliensis*, *Hylaeamys megacephalus* (Fischer, 1814)*, *Hylaeamys oniscus* (Thomas, 1904), Nectomys squamipes*, Oligoryzomys nigripes*, *Oxymycterus* sp.*, *Oxymycterus quaestor*, *Phyllomys* sp. Lund, 1839, *Proechimys guyannensis* (Geoffroy, 1803), Rattus norvegicus, Rhipidomys mastacalis*, Sooretamys angouya*, *Thaptomys nigrita*, *Trinomys dimidiatus*, *Trinomys setosus*	BA, ES, GO, MG, MS, PE, PR, RJ, RS, SC, SP	[104, 106, 110, 116, 118, 168, 170], this study*
*P. (P.) tripopsis* Guimarães, 1948	**Carnivora**: *Leopardus pardalis* **Cingulata**: *Dasypus novemcinctus* **Rodentia:** *Cerradomys subflavus*, *Echimys chrysurus*, *Euryoryzomys lamia*, *Holochilus brasiliensis*, *Hylaeamys megacephalus*, *Necromys lasiurus*, *Oligoryzomys nigripes*, *Oxymycterus dasytrichus*, *Rhipidomys mastacalis*	BA, CE, GO, MS, PE	[17, 103, 175]
*P. (P.) tripus* (Jordan, 1933)	**Didelphimorphia**: *Didelphis albiventris*, *Didelphis aurita*, *Didelphis marsupialis*, *Lutreolina crassicaudata, Monodelphis domestica* **Rodentia**: *Akodon cursor*, *Akodon montensis*, *Calomys expulsus*, *Calomys tener*, *Cavia aperea*, *Cerradomys subflavus*, *Euryzygomatomys spinosus*, Galea *spixii*, *Holochilus brasiliensis*, *Holochilus sciureus, Mus musculus brevirostris*, *Necromys lasiurus*, *Nectomys squamipes*, *Oligoryzomys nigripes*, *Oxymycterus dasytrichus*, *Rattus norvegicus*, *Rattus rattus alexandrinus*, *Rattus rattus frugivorus*, *Rhipidomys mastacalis*, *Thrichomys apereoides*, *Thrichomys inermis*, *Thrichomys laurentius*, *Trinomys albispinus*, *Trinomys setosus*, *Wiedomys pyrrhorhinos*	AL, BA, CE, ES, GO, MG, PE, PR, RJ, RN, SP	[17, 30, 33, 35, 110, 116, 120, 170, 173, 176]
**Tribe Rhopalopsyllini Oudemans, 1909**			
**Genus* Gephyropsylla Barrera, 1952***			
*G. klagesi klagesi* (Rothschild, 1904)	**Cingulata**: *Dasypus novemcinctus* **Didelphimorphia**: *Didelphis* spp., *Philander opossum* **Rodentia**: *Cerradomys subflavus*, *Proechimys guyannensis*, *Rhipidomys mastacalis*	AM, CE, GO, PA, RR	[17, 102, 177]
*G. klagesi samuelis* (Jordan & Rothschild, 1923)	**Didelphimorphia**: *Didelphis marsupialis* **Rodentia**: *Holochilus brasiliensis*, *Proechimys guyannensis*, *Proechimys longicaudatus* (Rengger, 1830)	AM, GO, RO, RR	[17, 102, 170]
**Genus* Hechtiella* Barrera, 1952**			
*H. lakoi* (Guimarães, 1948)	**Didelphimorphia**: *Philander opossum* **Rodentia**: *Euryoryzomys lamia*, *Oligoryzomys nigripes*, *Philander*, *Trinomys dimidiatus*, *Trinomys iheringi*	ES, MG, RJ, SP	[2, 17, 101, 169]
*H. lopesi* Guimarães & Linardi, 1993	**Rodentia**: *Proechymis* spp., *Trinomys iheringi*	SP	[2, 17, 99]
*H. nitidus* (Johnson, 1957)	**Cingulata**: *Dasypus novemcinctus* **Didelphimorphia**: *Didelphis marsupialis*, *Marmosops incanus*, *Metachirus nudicaudatus* **Rodentia**: *Necromys lasiurus*, *Nectomys squamipes*, *Trinomys dimidiatus*, *Trinomys iheringi*, *Trinomys paratus*	BA, ES, MG, RJ	[17, 35, 170, 176]
**Genus *Rhopalopsyllus* Baker, 1905**			
*R. australis australis* Rothschild, 1904	**Perissodactyla**: *Tapirus terrestris* **Rodentia**: *Dasyprocta fuliginosa* Wagler, 1832, *Proechimys guyannensis*	AP, PA, RO, RR	[17, 102, 160]
*R. australis tamoyus* Jordan & Rothschild, 1923	**Artiodactyla**: *Mazama rufa* Illiger, 1815 **Carnivora**: *Eira barbara*, *Nasua nasua*, *Procyon cancrivorus* **Cingulata:** *Dasypus novemcinctus* **Pilosa**: *Tamandua* spp. Gray, 1825 **Rodentia:** *Cuniculus paca*, *Dasyprocta azarae*, *Dasyprocta fuliginosa*	GO, MG, MS, MT, RO, SP	[2, 17, 103, 179]
*R. australis tupiniquinus* Guimarães, 1940	**Carnivora**: *Eira barbara*, *Leopardus pardalis*	SP	[2, 17]
*R. australis tupinus* Jordan & Rothschild, 1923	**Rodentia:** *Myoprocta acouchy* (Erxleben, 1777)	PA	[17]
*R. crypturi* Wagner, 1939	**Tinamiformes**: *Crypturellus obsoletus obsoletus*	SC	[17]
*R. garbei Guimarães, 1940*	**Rodentia**: *Myoprocta acouchy*	PA	[17]
*R. lugubris lugubris* Jordan & Rothschild, 1908	**Artiodactyla**: *Mazama americana* (Erxleben, 1777) **Cingulata:** *Dasypus novemcinctus* **Didelphimorphia**: *Didelphis marsupialis* **Rodentia**: *Akodon montensis*, *Cuniculus paca*, *Dasyprocta leporina*, *Oxymycterus quaestor*, *Trinomys dimidiatus*, *Trinomys iheringi*	ES, GO, MG, MS, MT, PA, RJ, RO, SC, SP	[17, 110, 103, 112, 178]
*R. lutzi lutzi* (Baker, 1904)	**Carnivora**: *Canis lupus familiaris*, *Cerdocyon thous*, *Leopardus pardalis*, *Nasua nasua*, *Puma yagouaroundi*, *Galictis vittata* **Cingulata:** *Dasypus novemcinctus* **Didelphimorphia**: *Didelphis albiventris*, *Didelphis aurita*, *Didelphis marsupialis*, *Philander opossum* **Pilosa:** *Tamandua tetradactyla* **Rodentia**: *Akodon serrensis*, *Dasyprocta azarae*, *Dasyprocta leporina*	BA, ES, GO, MG, MS, PR, RJ, RO, SP	[17, 33, 35, 103, 106, 108, 109, 110, 111, 113, 179]
*R. saevus* Jordan & Rothschild, 1923	**Cingulata**: *Dasypus novemcinctus* **Didelphimorphia**: *Didelphis marsupialis* **Rodentia**: *Dasyprocta fuliginosa*	MT, RO	[17, 178]
**Family Stephanocircidae Wagner, 1928**			
**Subfamily Craneopsyllinae Wagner, 1939**			
**Tribe Craneopsyllini Wagner, 1939**			
**Genus *Craneopsylla* Rothschild, 1911**			
*C. minerva minerva* (Rothschild, 1903)	**Chiroptera**: *Anoura geoffroyi geoffroyi* Gray, 1838, *Sturnira* (*Sturnira*) *lilium* (Geoffroy, 1810) **Didelphimorphia**: *Didelphis albiventris*, *Lutreolina crassicaudata*, *Marmosops incanus*, *Monodelphis domestica*, *Philander opossum* **Rodentia**: *Akodon cursor*, *Akodon montensis*, *Akodon reigi*, *Akodon serrensis*, *Brucepattersonius iheringi*, *Calomys tener*, *Cerradomys subflavus*, *Delomys dorsalis*, *Euryoryzomys lamia*, *Euryoryzomys russatus*, *Guerlinguetus aestuans*, *Holochilus sciureus*, *Necromys lasiurus*, *Nectomys squamipes*, *Oligoryzomys flavescens*, *Oligoryzomys nigripes*, *Oxymycterus dasytrichus*, *Oxymycterus quaestor*, *Proechimys guyannensis*, *Rattus rattus rattus*, *Rhipidomys mastacalis*, *Sooretamys angouya*, *Thaptomys nigrita*, *Trinomys dimidiatus*, *Wiedomys pyrrhorhinos*	AL, BA, CE, MG, PE, PR, RJ, RS, SC, SP	[17, 101, 116, 118, 171]
**Family Tungidae Taschenberg, 1880**			
**Subfamily Hectopsyllinae Baker, 1904**			
**Genus *Hectopsylla* Frauenfeld, 1860**			
*H. psittaci* Frauenfeld, 1860	**Columbiformes:** *Columba livia* Gmelin, 1789 **Passeriformes:** *Progne chalybea* Gmelin, 1789, *Turdus leucomelas* Vieillot, 1818	RJ, RS, SP	[17]
*Hectopsylla pulex* (Haller, 1880)	**Chiroptera**: *Histiotus velatus* (Geoffroy, 1824), *Molossus molossus* Pallas, 1766, *Molossus rufus* Geoffroy, 1805, *Peropteryx macrotis* (Wagner, 1843), *Phyllostomus hastatus*	BA, MG, PE, PR, RJ, RS, SC, SP	[17, 125, 128]
**Subfamily Tunginae Taschenberg, 1880**			
**Genus* Tunga* Jarocki, 1838**			
*T. bondari* Wagner, 1932	**Cariamiformes:** *Cariama cristata* (Temminck, 1823) **Pilosa:** *Tamandua tetradactyla*	BA, MG, SP	[17, 180]
*T. bossii* Avelar, Linhares & Linardi, 2012	**Rodentia:** *Delomys dorsalis*	RJ	[17, 131]
*T. caecata* (Enderlein, 1901)	**Rodentia**: *Mus musculus, Rattus rattus rattus, Rattus norvegicus, Akodon cursor, Necromys pixuna, Nectomys squamipes, Oligoryzomys nigripes, Oxymycterus sp., Rhypidomys mastacalis*	MG, PR, SP, RJ	[17, 181]
*T. hexalobulata* Avelar, Facury Filho & Linardi, 2013	**Artiodactyla**: * Bos taurus indicus*	MG	[131]
*T. penetrans* (Linnaeus, 1758)	**Artiodactyla**: *Bos taurus indicus*, *Sus scrofa* Linnaeus, 1758, *Capra hircus* Linnaeus, 1758, *Ovis aries* Linnaeus, 1758, *Pecari tajacu* Linnaeus, 1758 **Carnivora**: *Canis lupus familiaris, Felis cattus* Linnaeus, 1758, *Panthera onca* **Cingulata**: *Dasypus novemcinctus* **Passeriformes**: *Volatinia jacarina* (Linnaeus, 1766) **Perissodactyla**: *Equus caballus* Linnaeus, 1758, *Tapirus terrestris* **Pilosa**: *Tamandua tetradactyla, Myrmecophaga tridactyla* (Linnaeus, 1758) **Primata**: *Alouatta guariba clamitans* Cabrera, 1940, *Homo sapiens* **Rodentia**: *Cuniculus paca, Mus musculus*, *Rattus rattus rattus, Rattus norvegicus*	AC, AL, AM, AP, BA, CE, DF, ES, GO, MA, MG, MS, MT, PA, PB, PE, PI, PR, RJ, RN, RO, RS, RR, SC, SE, SP, TO	[17, 118, 132, 181, 182, 183, 184]
*T. terasma* Jordan, 1937	**Cingulata**: *Cabassous unicinctus* (Linnaeus, 1758), *Dasypus novemcinctus, Euphractus sexcinctus* (Linnaeus, 1758), *Priodontes maximus* (Kerr, 1792)	ES, GO, MA, MG, MS, SP	[17, 181, 185]
*T. travassosi* Pinto & Dreyfus, 1927	**Cingulata**: *Dasypus novemcinctus*	MG, SP	[17, 136, 181]
*T. trimamillata Pampiglione,* Trentini, Fioravanti, Onore & Rivasi, 2002	**Artiodactyla:** *Bos taurus indicus, Sus scrofa, Capra hircus*, *Ovis aries* **Primata**: *Homo sapiens* **Rodentia**: *Hydrochoerus hydrochaeris*	MG, SP	[181, 186, 187]

The species and locations highlighted in bold correspond to new occurrences. *Corresponds to samples collected in this study, AC, Acre; AL, Alagoas; AM, Amazonas; AP, Amapá; BA, Bahia; CE, Ceará; DF, Distrito Federal; ES, Espírito Santo; GO, Goiás; MA, Maranhão; MG, Minas Gerais; MS, Mato Grosso do Sul; MT, Mato Grosso; PA, Pará; PB, Paraíba; PE, Pernambuco; PI, Piauí; PR, Paraná; RJ, Rio de Janeiro; RN, Rio Grande do Norte; RO, Rondônia; RS, Rio Grande do Sul; RR, Roraima; SC, Santa Catarina; SE, Sergipe; SP, São Paulo; TO, Tocantins

### Family Leptopsyllidae Baker, 1904

Fleas of Leptopsyllidae are mainly found parasitizing rodents and are sometimes associated with synanthropic rodents, such as *Rattus rattus* Linnaeus, 1758, *R. norvegicus* (Berkenhout 1769), and *Mus musculus* Linnaeus, 1758, as well as wild rodents, but can also be found parasitizing lagomorphs and carnivores [41, 42]. In Brazil, this family is represented by a single genus called *Leptopsylla* Jordan & Rothschild 1911, which includes nine valid species, and just one is cosmopolitan and has already been recorded in Brazil—*Leptopsylla segnis* (Schönherr, 1811). The distribution of this species is related to the rodents present on ships, similar to what happens with the genus *Xenopsylla* Glinkiewicz 1907.

Some studies have reported that the species *L. segnis* is naturally infected with *Rickettsia typhi*, which parasitizes *R. rattus.* Experimental studies corroborated the vectorial potential of *L. segnis* for this pathogen, which is sometimes more effective than *X. cheopis* (Rothschild, 1903), as it settles for a long period in its host, favoring a high concentration of rickettsiae [43, 44]. However, this species has little importance because it rarely parasitizes humans [45]. In addition, *Bartonella* bacteria were also detected in this species [46].

Studies that focus on infectious agents of public health importance in members of this family are needed to elucidate the participation of these insects as vector, map the ecosystem where they are found, and avoid possible outbreaks.

### Family Pulicidae Billberg, 1820

Pulicidae is one of the most studied flea families in the world because it is considered a family of high relevance for medicine and veterinary medicine; it harbors generalist species that are able to infest humans and companion animals as well as act as vectors of pathogens [17]. This family includes approximately 21 genera divided into 4 subfamilies: Pulicinae Billberg, 1820; Xenopsyllinae Glienkiewicz, 1907; Archaeopsyllinae Oudemans, 1909; and Spylopsyllinae. The first three subfamilies can be found in Brazil [7, 17].

Nonetheless, Pulicidae and Tungidae were treated as a single group [7]. However, phylogenetic analyses have confirmed that Pulicidae is a monophyletic group distinct from Tungidae [9]. Further, Krasnov et al. [47] suggested that the Pulicidae and Leptopsyllidae families should be grouped on the basis of phylogenetic analyses, their origin, and migration in the Nearctic region. However, this new classification is still being studied.

The subfamily Pulicidae is represented by the tribes Pulicini and Echidnophagini, the latter being represented only by the genus *Echidnophaga* Olliff, 1886. In contrast, the first tribe has four genera: *Pulex* Linnaeus, 1758; *Delopsylla* Jordan, 1926; *Juxtapulex* Wagner, 1933; and *Moeopsylla* Rothschild, 1908 [13]. Of these, only the genus *Pulex* is cosmopolitan, and consequently, is also found in Brazil.

The genus *Echidnophaga* has been reported in several countries in South America, including Brazil, Chile, and Peru, and the only species found in these places is *Echidnophaga gallinacea* (Westwood, 1875). The adult female is known to have semipenetrating habits and is attached to the host through its mouthparts during feeding [48]. This species infests birds of the orders Galliformes [49], Anseriformes [50], and Columbiformes [51]. However, they can also be present in mammals such as rabbits [52], dogs [53], cats [53], rats [54] and wild carnivores [55].

These species can cause anemia, skin ulcers, and itching, and harbor several pathogens, such as *Rickettsia felis* and *R. asembonensis* [56], *Bartonella rochalimae* [57], fowl pox [58], and *Yersinia pestis* [59].

The genus *Pulex* is also commonly found in the Neotropics, but the only species reported in Brazil is *Pulex irritans* Linnaeus, 1758. This species can be associated with rodents, bats, and birds but prefers large wild and domestic mammals, such as dogs and cattle, and sometimes bites humans [60–62].

*Pulex irritans* is one of the first to be identified and one of the most studied species in terms of public health because it harbors several pathogens, such as *Bartonella* spp. [63] and *R. felis* [64]. As much as this species is susceptible to infection by *Y. pestis*, its vectorial competence is low and has already been proven through experimental infection, and it is unlikely that this species has any epidemiological relevance in the Black Plague cycle [65]. In addition, other pathogens have already been detected in this species, such as *Hymenolepis microstoma* Dujardin, 1845, and *Dipylidium caninum* Linnaeus, 1758, but the epidemiological importance of this species in the cycle of these endoparasites is not known [66].

The subfamily Xenopsyllinae is composed of seven genera that are distributed on the African and Asian continents, and the genus *Xenopsylla* Glienkiewicz, 1907, is one of greatest concerns in human medicine. In Brazil, two species have already been described: *X. cheopis* (Rothschild, 1903) and *X. brasiliensis* (Baker, 1904). Both species are believed to have originated from Africa and subsequently became cosmopolitan through rodents sheltered on ships during the colonization period [67, 68].

The species *X. cheopis*, considered the most efficient vector for *Y. pestis*, is frequently found in several regions of Brazil and parasitizes wild animals and synanthropic rodents [69, 70]. In addition, this bacterium can occur in two types of ecosystems: (1) in anthropized environments, with rodents of the genus *Rattus* and *Mus* that harbor the vector and disseminate the pathogen to companion animals and humans, this cycle is called the urban plague; and (2) in rural or wild environments, far from large cities, where the vector is maintained in small wild mammals (mainly rodents and marsupials), it is called the wild plague [14].

Inside the flea, the bacteria *Y. pestis* causes a proventricular block that prevents the feeding of the arthropod, with a regurgitation of blood in the place of a blood meal that carries the bacteria that infect the host [71, 72]. The absence of feeding by the vector results in aggressive behavior, with repeated attempts to feed on different hosts, favoring pathogen spread. This makes *Y. pestis* pathogenic for both vertebrate and invertebrate hosts, as a lack of food leads to flea dehydration, anorexia, and death [73–75].

In addition to *Y. pestis*, the flea *X. cheopis* also acts as a vector in the transmission cycle of murine typhus or endemic typhus caused by the bacterium *Rickettsia typhi*. This *Rickettsia* needs mammals and arthropod vectors to maintain itself in the environment, and unlike the *Y. pestis* cycle, this bacterium is transmitted through flea feces that are deposited on the host’s skin [76–78]. Once the flea acquires bacteria through feeding on infected mammals, *R. typhi* penetrates the intestinal epithelium, and after 10 days of infection, the insect is already capable of transmission. In addition to infecting this system, it also infects the muscle layer and reproductive organs, enabling transovarian perpetuation, which is not lethal to the invertebrate host or its progeny [79, 80].

The subfamily Archaeopsyllinae contains five genera, but the only one that occurs in Brazil, the genus *Ctenocephalides* Stiles & Collins, 1930, has two species recorded nationwide: *C. canis* (Curtis, 1826) and *C. felis felis* (Bouché, 1835), both of which are highly relevant in veterinary medicine. The species *C. canis* exhibits a more host-specific behavior, as it is predominantly found parasitizing only carnivores. Its occurrence is rare, and its distribution is primarily associated with temperate climate regions. [81–83]. On the contrary, the species *C. felis felis* is generalist and is able to parasitize a wide range of hosts, such as carnivores, xenarthrans, rodents, marsupials, primates, and lagomorphs [17, 84].

Allergic dermatitis syndrome to ectoparasite stings is a set of clinical signs commonly observed in dogs and cats, with alopecia in the hip region being the most common symptom caused by the species of *Ctenocephalides*. This syndrome is related only to flea bites but has recently been described in the context of parasitism by several ectoparasites and triggered by type I (immediate) and IV (late) hypersensitivity reactions to proteins present in the saliva of these arthropods [85, 86].

The flea *C. felis felis* has been found to be infected with the bacterium *Rickettsia felis*. This bacterium belongs to the spotted fever group, but in recent studies, it was reclassified into the transitional group through molecular data and phylogenetic analysis [87, 88]. Additionally, *Rickettsia felis* has already been detected in other species of fleas collected throughout Brazil [89] and its symptomatology in humans is still debatable. However, there are some reports of febrile episodes along with the development of a lesion at the site of the bite of the ectoparasite, and in more severe cases, it can cause neurological injuries such as encephalopathy, cerebral edema, and meningoencephalitis [90–92]. Other infectious agents that are related to cat fleas include *Bartonella* spp. [93], *D. caninum* [94], and *Dipetalonema reconditum* (Grassi, 1890) [95].

### Family Rhopalopsyllidae Oudemans, 1909

Among the eight families of order Siphonaptera that occur in Brazil, this family has the highest endemicity of species and can be considered the most important for the Brazilian territory. It is divided into two subfamilies, Rhopalopsyllinae Oudemans, 1909, and Parapsyllinae Enderlein, 1903, which are concentrated in neotropical regions. Furthermore, only the first is recorded in Brazil [96, 97].

The subfamily Rhopalopsyllinae comprises eight genera, five of which occur in Brazil: *Gephyropsylla* Barrera, 1952; *Hechtiella* Barrera, 1952*; Neotropsylla* Linardi & Guimarães, 1993*; Polygenis* Jordan, 1939; and *Rhopalopsyllus* Baker, 1905. The other three genera are (1) *Scolopsyllus*, which presents a single species, *S. columbianus* Mendez, 1968, described in Colombia as parasitizing rodents of the genus *Euryoryzomys* Weksler, Percequillo & Voss, 2006, and appears to be restricted to this locality [98]; (2) *Ayeshaepsylla* Smit, 1987, formerly considered *Polygenis*, and is also monotypic, having only *A. thurmani* Traub, 1972 described [99]; and (3) *Tiamastus* Jordan, 1939, which contains seven species distributed in several countries in South America, except Brazil [99].

The genus *Gephyropsylla* is rarely described in the literature, but it is related to parasitism in small hystricomorph rodents. This genus has just one species, *G. klagesi* (Rothschild, 1904), which is divided into three subspecies, all of which are recorded in neotropical regions. According to the literature, the center of dispersion of this species is in the territory of Venezuela, as it has the occurrence of all described subspecies [100]. The most generalist subspecies is *G. klagesi samuelis* (Jordan & Rothschild, 1923), which parasitizes species of the following orders: Rodentia, Didelphimorphia, Carnivora, Edentata, Chiroptera, Artiodactyla, and Sciuromorpha [17].

The genus *Hechtiella*, which parasitizes mainly rodents of the family Echimyidae Gray, 1825, has three species described in the Brazilian Atlantic Forest: *H. lakoi* (Guimarães, 1948)*, H. lopesi* Guimarães & Linardi, 1993, and *H. nitidus* (Johnson, 1957) [99, 101]; all parasitizing species of the genus *Proechimys* Allen, 1899 [99, 101].

The monotypic genus *Neotropsylla* [*Neotropsylla guimaraesi* (Linardi, 1978)] is considered endemic to the state of São Paulo, Brazil, and is associated with cricetid rodents [2, 99, 102].

The genus *Polygenis* has the largest number of species within the family Rhopalopsyllidae, and its distribution ranges from the southern tip of South America to the USA and is divided into two subgenera: *Polygenis* and *Neopolygenis*. Most species belonging to this genus are rodent parasites, but there are specimens collected that parasitize large mammals, demonstrating the low specificity of this genus in relation to its hosts [5, 103–105].

It is believed that the origin of the genus *Rhopalopsyllus* was within the Brazilian territory, as most species belonging to this genus occur here [106], as do their main hosts, i.e., rodents, marsupials, and xenarthrans. However, they can also occur in other wild mammals, such as coatis [*Nasua nasua* (Linnaeus, 1766)] and crab-eating foxes [*Cerdocyon thous* (Linnaeus, 1766)], and in domestic dogs [107–113]. The genus contains seven species and two subspecies, which are better elucidated in Table 1, as well as for all the other genera listed above.

Unlike the other genera found in this family, the genus *Polygenis* stands out. This genus can cause discomfort and itching and is also associated with several pathogens that are responsible for maintaining the wild plague (*Yersinia pestis*) circulating in forest environments via the use of wild rodents as amplifying hosts. Therefore, species of this genus are considered highly efficient vectors for this disease, proving to be even more efficient than the main vector, *Xenopsylla cheopis* [114–116]. Another pathogen that was recently associated with this genus is *Rickettsia felis*, which was initially detected in *Ctenocephalides felis felis*. [117], suggesting that this flea is capable of maintaining and transmitting *Rickettsia* in forest environments. In addition, Schott et al. [118] identified *Bartonella* sp. and *Rickettsia* sp. strain Taim, which showed phylogenetic proximity to *Rickettsia parkeri* in *Polygenis* spp.

Other infectious agents associated with fleas of the genus *Polygenis* include *Ehrlichia* sp. detected from *P.* (*P.*) *bohlsi bohlsi* (Wagner, 1901) in the Brazilian Pantanal region, which parasitizes *Trichomys* sp. Trouessart, 1880 [119]. Further, the nematode commonly found in rodents belonging to the genus *Hymenolepis* was also detected in fleas of the species *P.* (*P.*) *tripus* (Jordan, 1933), which is able to act as an intermediate host in its cycle [120].

### Family Stephanocircidae Wagner, 1928

This family is divided into two subfamilies: (1) Stephanocircinae, which comprises ectoparasite species of Australian marsupials; and (2) Craneopsyllinae Wagner, 1939, which is described as parasitizing marsupials and rodents in South America [17, 121].

The tribe Craneopsyllini (Craneopsyllinae) includes the genus *Craneopsylla* Rothschild, 1911, which is represented by a single species divided into two subspecies, namely, *C. minerva minerva* (Rothschild, 1903) and *C. minerva wolffhuegeli* (Rothschild, 1909). These species have been reported in several countries in South America, parasitizing rodents, marsupials, and bats, and only the subspecies *C. minerva minerva* was found in Brazil [31, 102, 122]. In addition, these fleas have been reported to be naturally infected with strains of the bacterial genera *Bartonella*, *Rickettsia*, and *Yersinia* [118].

In Brazil, *C. minerva minerva* has been recorded in the south, southeast, and northeast regions, parasitizing species of bats and a wide variety of wild rodents, including in regions where plague endemism has been recorded, albeit at low prevalence rates [17, 123].

### Family Tungidae Taschenberg, 1880

The Tungidae family is composed of two subfamilies, Hectopsyllinae Baker, 1904, and Tunginae Taschenberg, 1880, which are distributed in four genera and 23 species. The larvae of these fleas are easily found in the organic matter of sandy soils, which are commonly present in coastal regions or residential areas where houses are built with dirt floors [124].

The subfamily Hectopsyllinae is composed of two genera, *Rhynchopsyllus* Haller, 1880, and *Hectopsylla* Frauenfeld, 1860. The genus *Hectopsylla* can parasitize birds and mammals (mainly rodents and bats), and specimens of this genus only insert their mouthparts into the skin of their hosts, which is considered semipenetrating, and they have already been reported in Brazil and several other countries in South America [125, 126]. This genus is represented by the species *Hectopsylla psittaci* Frauenfeld, 1860, which is found in birds in southern and southeastern Brazil [17].

Like the previous genus, the genus *Rhynchopsyllus* also has a semipenetrating habit and is found parasitizing several species of bats in South America, occurring exclusively in the neotropical region, and only the species *R. pulex* Haller, 1880, has been recorded in Brazil [127, 128]. Records of the Rhybchopsyllus paraziting birds and rodents were incidental [129].

There are two genera in the subfamily Tunginae: (1) *Neotunga* Smit, 1962, with the type species *Neotunga euloidea* Smit, 1962 recorded parasitizing placental mammals of the order Pholidota on the African continent [24], and (2) the important genus *Tunga* Jarocki, 1838, which is represented by species with penetrating habits when in contact with the host, where the female flea will insert part of its body into its epidermis, leaving the last two abdominal segments in contact with the external environment, making it possible to visualize only the genital pore and its respiratory stigma. After this process, the flea begins to feed, and it is possible to verify hypertrophy of the body, forming a neosome of 5–13 mm. After the peak of engorgement, the ectoparasite begins oviposition, releasing the eggs directly into the environment [11].

This genus occurs mainly in neotropical regions because 9 of the 13 species are distributed in South America, and one species, *Tunga penetrans* (Linnaeus, 1758), also occurs in this portion of the African continent [130]. Additionally, this genus can be found parasitizing humans, dogs, cats, cattle, pigs, goats, sheep, xenarthrans, and rodents and accidentally parasitizing elephants and primates [131–133].

Currently, the species of the genus *Tunga* are divided into “Group *penetrans*” (*T. penetrans*, *T. trimamillata* Pampiglione, Trentini, Fioravanti, Onore & Rivasi, 2002, *T. hexalobulata* Avelar, Facury Filho & Linardi, 2013, *T. travassosi* Pinto & Dreyfus, 1927, *T. bondari* Wagner, 1932, and *T. terasma* Jordan, 1937), which are associated with mainly parasitizing xenarthrans, and six of them are recorded in Brazil; and “Grupo *caecata*” [*T. caecata* (Enderlein, 1901), *T. caecigena* Jordan & Rothschild, 1921, *T. callida* Li & Chin, 1957, *T. libis* Smit, 1962, *T. monositus* Barnes & Radovsky, 1969, *T. bossii* Avelar, Linhares & Linardi, 2012, and *T. bonneti* Beaucournu & González-Acuña, 2012], with species that parasitize only rodents, and two occurring in the Brazilian territory [131, 134]. This division into two groups is based on morphological differences, geographical distributions, and differences in hosts [135–139]. Table 1 presents the eight species that occur in Brazil, with their respective locality records.

The species *Tunga penetrans* has the widest distribution within the genus, occurring throughout the neotropical region in sub-Saharan Africa, with greater relevance in populations in a situation of socioeconomic vulnerability [7, 140–144].

Tungiasis is a condition caused by females of *T. penetrans* and *T. trimamillata*. A female of this species inserted into the host’s skin can cause discomfort, itching, and even local ulcerations, depending on the level of infestation [145]. In addition, these injuries caused by female engorgement can cause nail and tegument deformities, difficulty in locomotion, or even secondary infections that can culminate in the death of the host due to complications [146–148].

### New records

A total of 211 fleas were taxonomically identified and separated into 3 genera, and 7 species were identified (Table 2). Within the Rhopalopsyllidae family, the genus *Polygenis*, which interacts with rodents and marsupials, was collected. Three species from this genus have been documented: *Polygenis (Polygenis) bohlsi jordani* (Lima, 1937), *Polygenis (Polygenis) rimatus* (Jordan, 1932), and *Polygenis (Polygenis) roberti roberti* (Rothschild, 1905). Here, we register the first encounter of *P. (P.) bohlsi jordani* in the state of São Paulo and present new host associations highlighted in Table 1. The other two species had already been recorded at the collection state, but new host interactions were also observed here.

**Table 2:** Slide-mounted flea specimens collected on small mammals in a preserved ecological area (Legado das Águas—Reserva Votorantim) inside the Atlantic Rainforest Biome, during the years 2018–2021

Host	Flea species							Total
	*Adoratopsylla (A.) antiquorum antiquorum*	*Adoratopsylla (T.) intermedia intermedia*	*Adoratopsylla (T.) sinuata*	*Hormopsylla fosteri*	*Polygenis (P.) bohlsi jordani*	*Polygenis (P.) rimatus*	*Polygenis (P.) roberti roberti*	
Chiroptera								
*Chrotopterus auritus*							1F	1
*Nyctinomops sp.*				1M, 4F				5
Didelphimorphia								
*Didelphis aurita*		1F					4M, 3F	8
*Gracilinanus sp.*							1M, 1F	2
*Metachirus nudicaudatus*	2M, 2F	1F	6M, 2F		2F	2F	10M, 10F	37
*Monodelphis sp.*			1M				1M, 1F	3
Rodentia								
*Akodon sp.*						1F	2M, 1F	4
*Brucepattersonius sp.*					1F		4M	5
*Euryoryzomys russatus*	1M, 1F	1M, 1F	1M		12F	2 M, 2F	34M, 37F	92
*Guerlinguetus sp.*							1M, 1F	2
*Holochilus sp.*							4M, 2F	6
*Hylaeamys sp.*							5M, 2F	7
*Nectomys squamipes*							3M, 7F	10
*Oligoryzomys sp.*					1F		1M, 4F	6
*Oxymycterus sp.*	1F				2F	2 M, 1F	4F	10
*Phyllomys sp.*							2M, 1F	3
*Rhipidomys sp.*							5M, 1F	6
*Sooretamys sp.*							1M, 2F	3
*Thaptomys sp.*	1F							1
Total	8	4	10	5	18	10	156	211

**Fig. 2:**
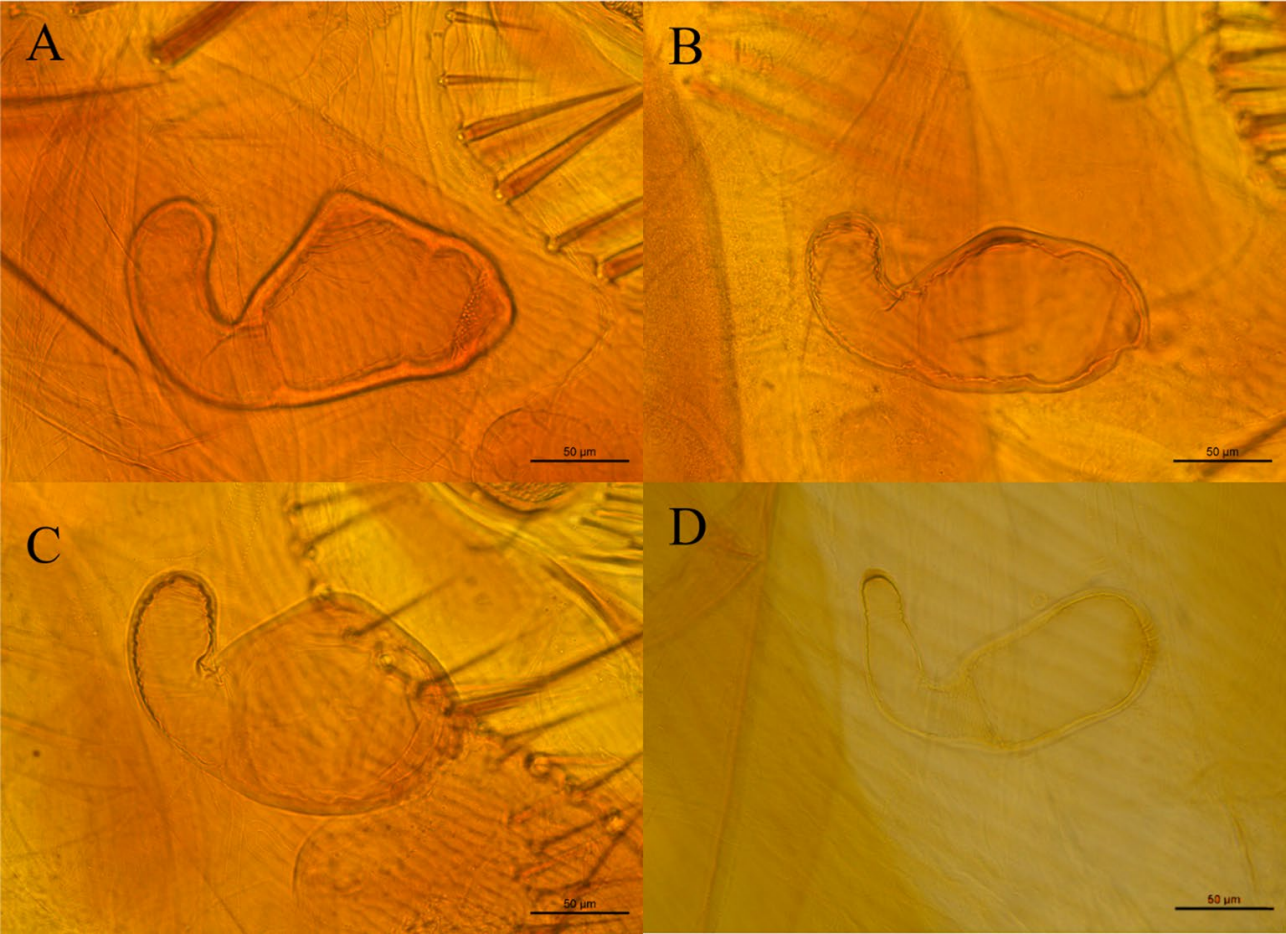
Spermathecae of the collected female fleas; **A** *Polygenis* (*Polygenis*) *roberti roberti*; **B** *Polygenis* (*Polygenis*) *rimatus*; **C** *Polygenis* (*Polygenis*) *bohlsi jordani*; **D** *Adoratopsylla* (*Tritopsylla*) *intermedia intermedia*

**Fig. 3:**
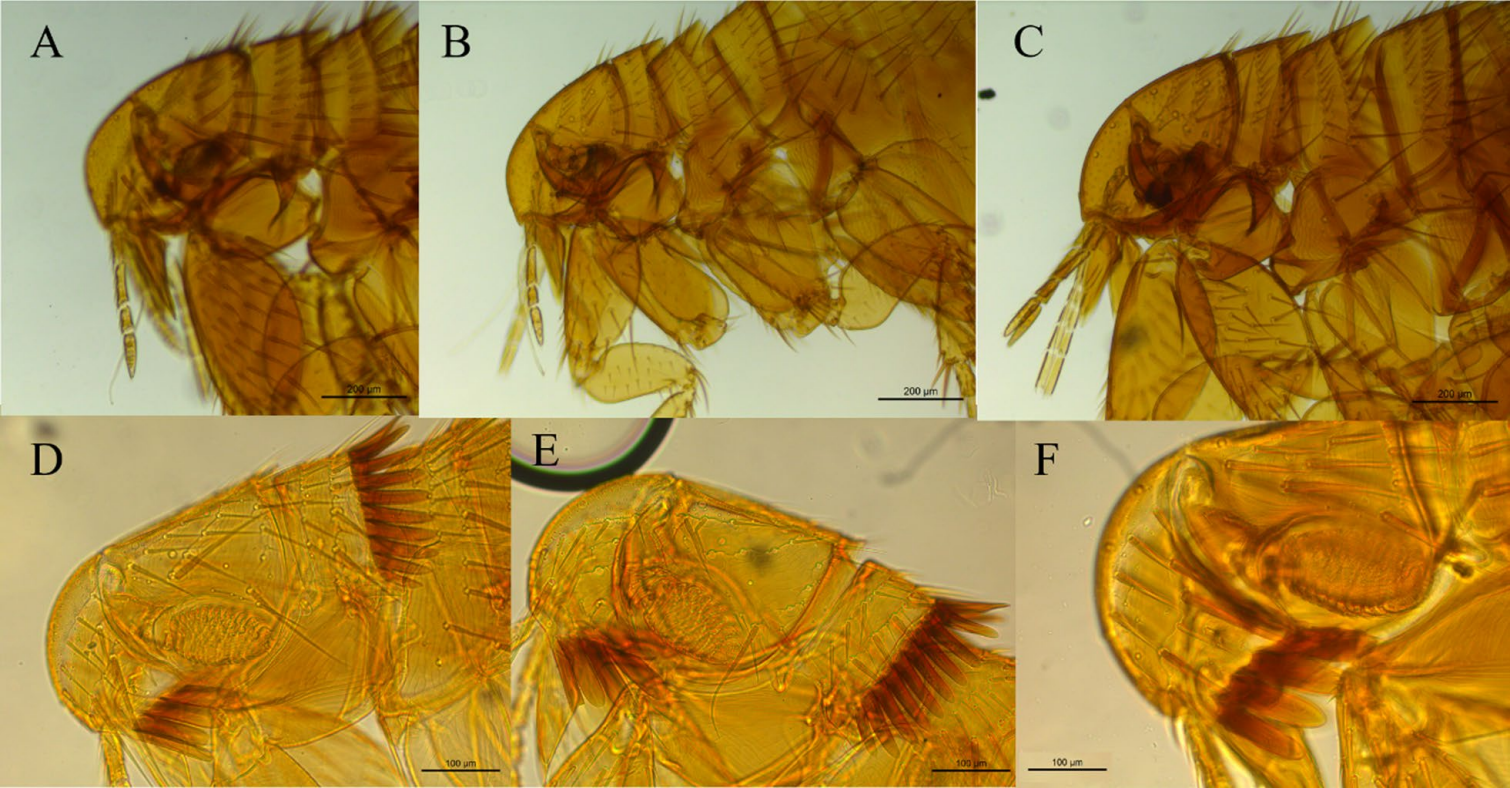
Morphology of the head of the collected fleas. **A** *Polygenis* (*Polygenis*) *roberti roberti*; **B** *Polygenis* (*Polygenis*) *rimatus*; **C** *Polygenis* (*Polygenis*) *bohlsi jordani*; **D** *Adoratopsylla* (*Adoratopsylla*) *antiquorum antiquorum*; **E** *Adoratopsylla* (*Tritopsylla*) *intermedia intermedia*; **F** *Adoratopsylla* (*Tritopsylla*) *sinuata*

**Fig. 4:**
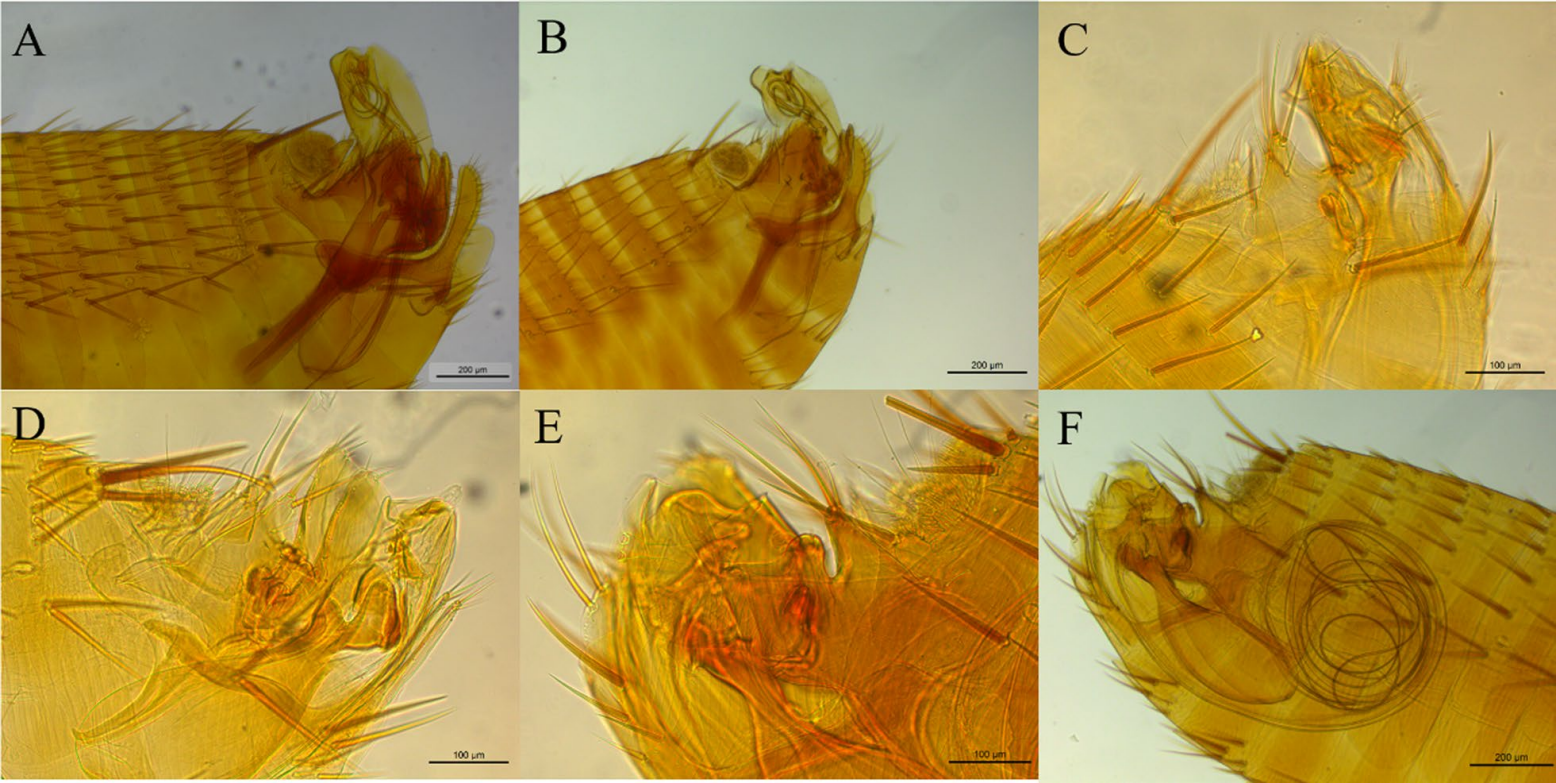
Morphology of aedagus and claspers of collected male fleas. **A** *Polygenis* (*Polygenis*) *rimatus*; **B** *Polygenis* (*Polygenis*) *roberti roberti*; **C** *Adoratopsylla* (*Adoratopsylla*) *antiquorum antiquorum*; **D** *Adoratopsylla* (*Tritopsylla*) *intermedia intermedia*; **E, F** *Adoratopsylla* (*Tritopsylla*) *sinuata*

The other samples belong to the Ctenophtalmidae family with three representatives: *Adoratopsylla (Adoratopsylla) antiquorum antiquorum* (Rothschild, 1904), *Adoratopsylla (Tritopsylla) intermedia intermedia* (Wagner, 1901), and *Adoratopsylla (Tritopsylla) sinuata* (Guimarães, 1945). All the species identified present new host associations, and *A. (T.) sinuata*, which had been previously described only in the state of Paraná, was also found in this study, resulting in a new locality record. All the new records are noted in Table 1. The images taken from the collected fleas are presented in Figs. 2, 3, and 4.

**Fig. 5:**
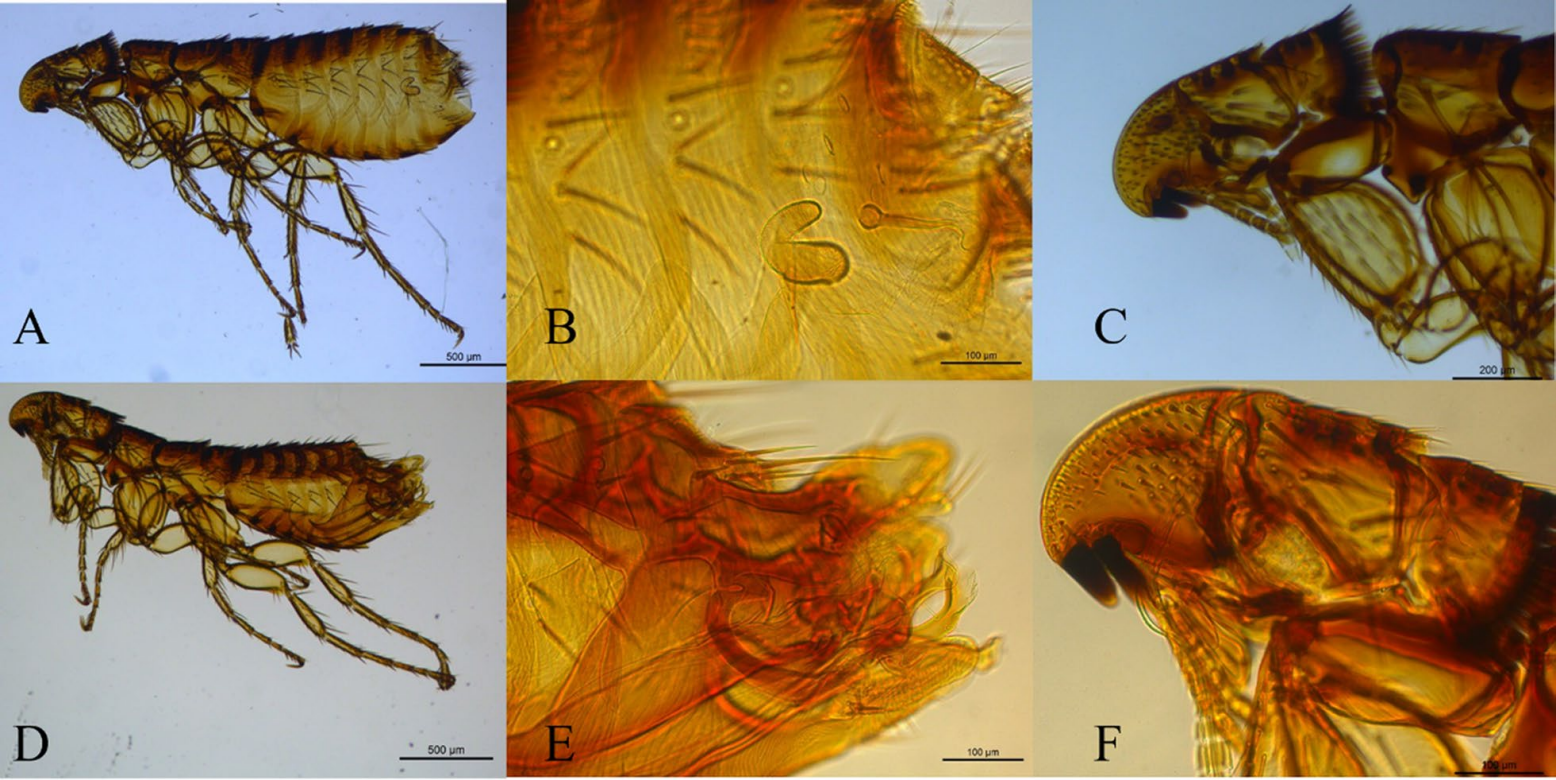
Morphology of *Hormopsylla fosteri*. **A** Female; **B** Spermathecae; **C** head of the female; **D** male; **E** clasper and aedagus; **F** head of the male

Two species were recovered from the sampled bats: one female of *P. (P.) roberti roberti* from *Chrotopterus auritus* (Peters, 1856) and five specimens of *Hormopsylla fosteri* (Rothschild, 1903) from three *Nyctinomops* sp. Miller, 1902. This is the first time that the species *P. (P.) roberti roberti* has been found to interact with bats, making this a new record. The genus *Hormopsylla* belongs to the Ischnopsyllidae family, which is known for having a specific association with bats (Fig. 5). All new records of hosts and localities highlight the importance of further studies on the flea fauna in Brazil, given the limited research on the diversity and collection of new specimens in Brazilian biomes. Moreover, these findings pave the way for future studies in other Brazilian states, as the present study was restricted to the Atlantic Forest biome in the state of São Paulo. All the identified fleas are presented in Table 2.

### Molecular analysis

A total of 105 flea samples were processed individually through molecular tools, belonging to the following species: *P. (P.) roberti roberti*, *P. (P.) bohlsi jordani, P. (P.) rimatus*, *A. (A.) antiquorum antiquorum, A. (T.) intermedia intermedia*, and *A. (T.) sinuata*. All the samples produced the expected amplicons when subjected to COII analysis, validating the extraction procedures. After that, all the samples were screened for rickettsial DNA as previously described, but none amplified the pathogen gene, which is an expected result since *Rickettsia* species associated with fleas are more commonly found in cosmopolitan fleas [89, 93] rather than endemic species. Schott et al. [118] detected the presence of *Rickettsia* in fleas belonging to the genera *Polygenis* and *Craneopsylla*, but only specimens collected in the Pampa Biome; the samples collected in the Atlantic Rainforest Biome turned out to be negative as well. *Rickettsia* species were also detected by Berrizbeitia et al. [149] and López-Pérez et al. [56], however, the fleas they sampled originated from a different biome than ours and were represented either by species that we did not collect or by cosmopolitan species, respectively.

## Conclusions

Fleas are highly important in human health and veterinary medicine, as in addition to causing physical discomfort due to their blood meal, they are also capable of transmitting various pathogens that can cause great damage to the vertebrate host.

Studies involving fleas are limited and focused on a few leading families already known, disregarding the lesser-known families that are incredibly scarce, demonstrating the need for further analyses evaluating not only the epidemiology of fleas as parasites and the detection of zoonotic pathogens, but also expanding the knowledge about the classifications and subclassifications of these invertebrates, reducing the gaps in knowledge about the characteristics of the species and subgroups, thus making possible a greater association with the epidemiology of infestations and related comorbidities through a more accurate knowledge of the real diversity of the order Siphonaptera. This study showed new locality reports and new hosts associations, including the screening for *Rickettsia* bacteria. Even though the results were negative, they open space for new research in anthropized environments, allowing us to compare how human activity influences the epidemiological cycle of vector-borne diseases.

## Supplementary Information

Additional file 1

## Data Availability

No datasets were generated or analyzed during the current study.
