# Supplementary material for: Brazilian fleas (Hexapoda: Siphonaptera): diversity, host associations, and new records on small mammals from the Atlantic Rainforest, including Rickettsia screening
Source: Parasit Vectors. 2025 Apr 4;18:130. doi: 10.1186/s13071-025-06755-6 (PMC11969852; doi:10.1186/s13071-025-06755-6)
Supplement: Supplementary file 1 — Additional file 1 [file 13071_2025_6755_MOESM1_ESM.docx]

Additional file 1: Table S1: Flea species with state locality and hosts described in Brazil, including new records of fleas collected from a preserved Atlantic Rainforest reserve (Legado das Águas – Reserva Votorantim) from 2018 to 2021.

| **Taxon** | **Host** | **Brazilian State** | **Reference** |
| --- | --- | --- | --- |
|  |  |  |  |
| **Family Ceratophyllidae Dampf, 1908** | | | |
| **Genus Nosopsyllus Jordan, 1933** | | | |
| *N. fasciatus* (Bosc, 1800) | **Carnivora**: *Canis lupus familiaris* Linnaeus, 1758.  **Rodentia**: *Mus musculus* brevirostris (Waterhouse, 1837), *Rattus norvegicus* (Berkenhout, 1769), *Rattus rattus alexandrinus* (Geoffroy, 1803), *Rattus rattus rattus* (Linnaeus, 1758). | GO, RJ, SP | Linardi and Guimaraes, 2000 |
| **Family Ctenophthalmidae Rothschild, 1915** |  |  |  |
| **Subfamily Doratopsyllinae Wagner, 1939** |  |  |  |
| **Tribe Tritopsyllini Cunha, 1914** |  |  |  |
| **Genus *Adoratopsylla* Ewing, 1925** |  |  |  |
| **Subgenus *Adoratopsylla* Ewing, 1925** |  |  |  |
| *A. (A.) antiquorum antiquorum* (Rothschild, 1904) | **Carnivora:** *Puma yagouaroundi* (Geoffroy, 1803).  **Didelphimorphia:** *Didelphis albiventris* Lund, 1840, *Didelphis aurita* (Wied-Neuwied, 1826), *Didelphis marsupialis* Linnaeus, 1758, *Marmosa (Micoureus) paraguayanus* (Tate, 1931), *Marmosa murina* (Linnaeus, 1758), *Marmosops incanus* (Lund, 1840), *Marmosops parvidens* (Tate, 1931), *Metachirus nudicaudatus* (Desmarest, 1817)*, *Monodelphis americana* (Müller, 1776), *Monodelphis dimidiata* (Wagner, 1847), *Monodelphis domestica* (Wagner, 1842), *Monodelphis (Microdelphys) iheringi (*Thomas, 1888), *Philander opossum* (Linnaeus, 1758).  **Rodentia:** *Akodon cursor* (Winge, 1887), *Akodon montensis* Thomas, 1913, *Akodon serrensis* Thomas, 1902, *Cerradomys subflavus* (Wagner, 1842), *Delomys dorsalis* (Hensel, 1873), *Delomys sublineatus* (Thomas, 1903), *Euryoryzomys russatus* (Wagner, 1848)*, *Galea spixii* (Wagler, 1831), *Necromys lasiurus* (Lund, 1841), *Nectomys squamipes* (Brants, 1827), *Oligoryzomys moojeni* Weksler & Bonvicino, 2005, *Oligoryzomys nigripes* (Olfers, 1818), *Oxymycterus* sp. Waterhouse, 1837*, *Rhipidomys mastacalis* (Lund, 1840), *Thaptomys nigrita* (Lichtenstein, 1829)*, *Trinomys setosus* (Desmarest, 1817). | AL, BA, CE, ES, MG, PR, RJ, SP* | Barros et al., 1993; Barros-Battesti and Arzua, 1997; Linardi and Guimaraes, 2000; Horta et al., 2007; Pinto et al., 2009; Oliveira et al., 2010; **this study*** |
| *A. (A.) antiquorum ronnai* Guimarães, 1954 | **Didelphimorphia:** *Marmosa (Micoureus) paraguayanus*, *Philander opossum.* | RS, SC, SP | Linardi and Guimaraes, 2000 |
| *A. (A.) bisetosa* Ewing, 1925 | **Didelphimorphia:** *Monodelphis brevicaudata* (Erxleben, 1777). | AM | Linardi and Guimaraes, 2000 |
| Subgenus *Tritopsylla* Cunha, 1914 |  |  |  |
| *A. (T.) intermedia intermedia* (Wagner, 1901) | **Carnivora:** *Cerdocyon thous* (Linnaeus, 1766), *Procyon cancrivorus* Cuvier, 1798.  **Didelphimorphia:** Chironectes minimus (Zimmermann, 1780), *Didelphis albiventris*, *Didelphis aurita**, *Lutreolina crassicaudata* (Desmarest, 1804), *Marmosa (Marmosa) murina* (Linnaeus, 1758), *Marmosa (Micoureus) paraguayanus, Marmosops incanus*, *Metachirus nudicaudatus**, *Monodelphis americana*, *Monodelphis (Microdelphys) iheringi*, *Philander opossum.*  **Rodentia:** *Cavia aperea* Erxleben, 1777, *Euryoryzomys russatus**, *Guerlinguetus* sp. Gray, 1821, *Nectomys squamipes*, *Trinomys paratus* (Moojen, 1948). | BA, ES, MG, PA, PR, RJ, SC, SP* | Barros-Battesti and Arzua, 1997; Linardi and Guimarães, 2000; Salvador et al., 2007; Pinto et al., 2009; Oliveira et al., 2010; **this study*** |
| *A. (T.) sinuata* Guimarães, 1945 | **Didelphimorphia:** *Metachirus nudicaudatus**, *Monodelphis* sp. Burnett, 1830*, *Philander opossum*.  **Rodentia: *Euryoryzomys russatus*.** | PR, SP* | Linardi and Guimarães, 2000; **this study*** |
| **Family Ischnopsyllidae Tiraboschi, 1904** |  |  |  |
| **Subfamily Ischnopsyllinae Wahlgren, 1907** |  |  |  |
| **Tribe Ischnopsyllini Wahlgren, 1907** |  |  |  |
| **Genus *Myodopsylla* Jordan & Rothschild, 1911** |  |  |  |
| *M. wolffsohni wolffsohni* (Rothschild, 1903) | **Chiroptera:** *Eptesicus* sp. Rafinesque, 1820, *Molossus currentium currentium* Thomas, 1901, *Myotis levis* (Geoffroy, 1824), *Myotis nigricans nigricans* (Schinz, 1821), *Noctilio leporinus* Linneus, 1758. | AL, AM, MT, PR, SC | Linardi and Guimaraes, 2000; Arzua et al., 2002 |
| **Tribe Sternopsyllini Medvedev, 1985** |  |  |  |
| **Genus *Hormopsylla* Jordan & Rothschild, 1921** |  |  |  |
| *H. fosteri* (Rothschild, 1903) | **Chiroptera:** *Cynomops abrasus abrasus* (Temminck, 1827), *Desmodus rotundus* (Geoffroy, 1810), *Lasiurus (Lasiurus) blossevilli* blossevilli (Lesson, 1826), *Nyctinomops* sp. Miller, 1902*, *Nyctinomops laticaudatus* (Geoffroy, 1805), *Phyllostomus hastatus* Pallas, 1767. | MG, PB, RN, SP | Rodriguez et al., 1999; Linardi and Guimaraes, 2000; **this study*** |
| **Genus *Ptilopsylla* Jordan & Rothschild, 1921** |  |  |  |
| *P. leptina* Jordan & Rothschild, 1921 | **Chiroptera:** *Noctilio albiventris* Desmarest, 1818, *Nyctinomops laticaudatus europs* (Allen, 1889). | MS | Linardi and Guimaraes, 2000 |
| **Genus *Rothschildopsylla* Guimarães, 1953** |  |  |  |
| *R. noctilionis* (Costa Lima, 1920) | **Chiroptera:** *Noctilio albiventris.* | MS | Linardi and Guimaraes, 2000; Linardi, 2017 |
| **Genus *Sternopsylla* Jordan & Rothschild, 1921** |  |  |  |
| *S. distincta distincta* (Jordan & Rothschild, 1921) | **Chiroptera:** *Molossus currentium* *currentium,* *Nyctinomops laticaudatus*, *Tadarida brasiliensis* (Geoffroy, 1824) | MG, PR, RS | Linardi and Guimaraes, 2000 |
| **Family Leptopsyllidae Baker, 1904** |  |  |  |
| **Subfamily Leptopsyllinae Baker, 1904** |  |  |  |
| **Genus *Leptopsylla* Jordan & Rothschild, 1911** |  |  |  |
| *L. segnis* (Schönherr, 1811) | **Didelphimorphia:** *Didelphis aurita*.  **Rodentia:** *Cerradomys* *subflavus*, *Mus* *musculus* Linnaeus, 1758, *Oxymycterus* *delator* Thomas, 1903, *Rattus* *norvegicus*, *Rattus* *rattus* *alexandrinus*, *Rattus* rattus *frugivorus* (Rafinesque, 1814), *Rattus* *rattus* *rattus*. | MG, PE, RS, SC, SP | Linardi and Guimaraes, 2000; Salvador et al., 2007; Winkel et al., 2014 |
| **Family Pulicidae Billberg, 1820** |  |  |  |
| **Tribe Archaeopsyllini Oudemans, 1909** |  |  |  |
| **Genus *Ctenocephalides* Stiles & Collins, 1930** |  |  |  |
| *C. canis* (Curtis, 1826) | **Carnivora**: *Canis lupus familiaris*, *Cerdocyon thous,* *Felis catus* Linnaeus, 1758. | AM, BA, MG, PE, PR, RJ, RS, SC, SP | Cerqueira et al., 2000; Linardi and Guimaraes, 2000; Horta et al., 2006; Guimarães et al., 2011; Silva et al., 2017; Oliveira et al., 2021 |
| *C. felis felis* (Bouché, 1835) | **Artiodactyla:** *Blastocerus dichotomus* Illiger, 1815, *Bos taurus indicus* (Linnaeus, 1758).  **Carnivora**: *Canis lúpus familiaris* Linnaeus, 1758, *Cerdocyon* *thous*, *Chrysocyon* *brachyurus* (Illiger, 1815), *Eira* *barbara* (Linnaeus, 1758), *Felis catus*, *Leopardus* *pardalis* (Linnaeus, 1758), *Leopardus* *tigrinus* (Schreber, 1775), *Lycalopex* *vetulus* (Lund, 1842), *Nasua* *nasua* (Linnaeus, 1766), *Panthera* *onca* (Linnaeus, 1758), *Procyon* *cancrivorus*, *Puma yagouaroundi*.  **Cingulata:** *Dasypus* *novemcinctus* Linnaeus, 1758.  **Didelphimorphia:** *Didelphis* *albiventris*, *Didelphis* *aurita*, *Didelphis* *marsupialis*, *Lutreolina* *crassicaudata,* *Marmosa* (*Micoureus*) *paraguayanus*, *Monodelphis* *domestica*.  **Lagomorpha:** *Sylvilagus* *brasiliensis* (Linnaeus, 1758).  **Pilosa:** *Tamandua tetradactyla* (Linnaeus, 1758).  **Perissodactyla:** *Tapirus terrestris* (Linnaeus, 1758).  **Primata:** *Homo sapiens* (Linnaeus, 1758), *Sapajus nigritus* (Goldfuss 1809).  **Rodentia:** *Akodon* *serrensis*, *Cavia porcellus* (Linnaeus, 1758), *Cerradomys* *subflavus*, *Euryzygomatomys* *spinosus* (G. Fischer, 1814), *Galea* *spixii*, *Guerlinguetus* *aestuans* (Linnaeus, 1766), *Hydrochoerus* *hydrochaeris* (Linnaeus, 1766), *Necromys* lasiurus, *Oligoryzomys* *nigripes*, *Oxymycterus* *dasytrichus* (Schinz, 1821), *Oxymycterus* *delator*, *Thrichomys* *laurentius* (Thomas, 1904), *Trinomys* *albispinus* (Geoffroy, 1838). | AL, AM, AP, BA, CE, ES, DF, GO, MG, MS, MT, PA, PB, PE, PI, PR, RJ, RN, RS, RO, RR, SC, SP, TO | Barros-Battesti and Arzua, 1997; Linardi and Guimarães, 2000; Szabó et al., 2000; Pinto et al., 2009; Mendes-de-Almeida et al., 2011; Heukelbach et al., 2012; Horta et al., 2014; Paz et al., 2015; Regolin et al., 2015; Schott et al., 2019; Paz et al., 2022; Gonçalves et al., 2023; Silva et al., 2023 |
| **Tribe Pulicini Billberg, 1820** |  |  |  |
| **Genus *Pulex* Linnaeus, 1758** |  |  |  |
| *P. irritans* Linnaeus, 1758 | **Carnivora**: *Canis lupus familiaris*, *Cerdocyon* *thous*, *Conepatus* *chinga* (Molina, 1782), *Chrysocyon brachyurus*, *Galictis* *vittata* (Schreber, 1776), *Leopardus geoffroyi* (d'Orbigny & Gervais, 1844), *Leopardus* *pardalis*, *Panthera* *onca*, *Procyon* *cancrivorus*.  **Chiroptera:** *Nyctinomops* *laticaudatus*.  **Pilosa:** *Tamandua tetradactyla*.  **Primata:** *Homo sapiens*.  **Didelphimorphia:** *Monodelphis* *domestica*, *Philander* *opossum*.  **Rodentia:** *Cuniculus* *paca* (Linnaeus, 1766), *Galea* *spixii*, *Guerlinguetus* *aestuans*, *Holochilus* *brasiliensis* (Desmarest, 1819), *Kerodon* *rupestris* (Wied-Neuwied, 1820), *Oligoryzomys* *nigripes*, *Thrichomys* *inermis* (Pictet, 1841), *Thrichomys* *laurentius*, *Trinomys* *dimidiatus* (Günther, 1877), *Trinomys* *setosus*, *Wiedomys* *pyrrhorhinos* (Wied-Neuwied, 1821). | AL, BA, CE, ES, GO, MG, PA, PB, PE, PI, PR, RJ, RN, RS, SC, SP | Linardi and Guimarães, 2000; Carvalho et al., 2001; Curi et al., 2010; Pereira et al., 2014; Santos et al., 2016; Fontalvo et al., 2017; Souza et al., 2021; |
| **Tribe Xenopsyllini Glienkiewicz, 1907** |  |  |  |
| **Genus *Xenopsylla* Glienkiewicz, 1907** |  |  |  |
| *X. brasiliensis* (Baker, 1904) | **Carnivora**: *Canis lupus familiaris*.  **Rodentia:** *Necromys lasiurus*, *Oligoryzomys nigripes*, *Mus* *musculus, Rattus* *norvegicus*, *Rattus* *rattus* *alexandrinus*, *Rattus* rattus *frugivorus*, *Rattus* *rattus* *rattus*. | CE, PB, PE, RN, RJ, RS, SP | Linardi and Guimarães, 2000 |
| *X. cheopis* (Rothschild, 1903) | **Carnivora**: *Canis lupus familiaris*, *Cerdocyon* *thous*.  **Didelphimorphia:** *Didelphis* *aurita*, *Didelphis* *marsupialis*, *Monodelphis* *domestica.*  **Rodentia:** *Akodon* *cursor*, *Akodon* *montensis*, *Cavia* *aperea*, *Cerradomys* *subflavus*, *Galea* *spixii*, *Holochilus* *brasiliensis*, *Holochilus* *sciureus* Wagner, 1842, *Necromys* *lasiurus*, *Nectomys* *squamipes*, *Rattus* *rattus* *rattus*, *Thrichomys* *apereoides* (Lund, 1839), *Thrichomys* *inermis*, *Thrichomys* *laurentius*, *Trinomys* *elegans* (Lund, 1841), *Trinomys* *setosus.* | AL, BA, CE, MG, PE, PR, RJ, RN, RS, SC, SP | Cerqueira et al., 2000; Linardi and Guimarães, 2000; Carvalho et al., 2001; Ribeiro et al., 2003; Oliveira et al., 2009; Porta et al., 2014; Bezerra-Santos et al., 2020 |
| **Family Rhopalopsyllidae Oudemans, 1909** |  |  |  |
| **Subfamily Rhopalopsyllinae Oudemans, 1909** |  |  |  |
| **Tribe Polygenini Linardi & Guimarães, 1993** |  |  |  |
| **Genus *Neotropsylla* Linardi & Guimarães, 1993** |  |  |  |
| *N. guimaraesi* (Linardi, 1978) | **Rodentia:** *Calomys* sp. Waterhouse, 1837 | SP | Linardi and Guimarães, 2000; Linardi, 2011 |
| **Genus *Polygenis* Jordan, 1939** |  |  |  |
| **Subgenus *Polygenis* (*Neopolygenis*) Linardi & Guimarães, 1993** |  |  |  |
| *P. (N.) atopus* (Jordan & Rothschild, 1922) | **Carnivora**: *Eira barbara, Felis catus, Procyon cancrivorus.*  **Didelphimorphia:** *Didelphis* *albiventris*, *Didelphis* *aurita*, *Didelphis* *marsupialis*, *Philander* *opossum*.  **Passeriformes:** *Haplospiza* *unicolor* Linnaeus, 1766.  **Rodentia:** *Akodon* *cursor*, *Akodon* *montensis*, *Caluromys* philander (Linnaeus, 1758), *Cerradomys* *subflavus*, *Delomys* *dorsalis*, *Euryoryzomys* *russatus*, *Guerlinguetus* sp., *Holochilus* *brasiliensis*, *Nectomys* *squamipes*, *Oligoryzomys* *flavescens* (Waterhouse, 1837), *Oligoryzomys* *nigripes*, *Oxymycterus* *dasytrichus*, *Rhipidomys* *mastacalis*, *Sooretamys* *angouya* (Fischer, 1814). | MG, PR, RS, RJ, SC, SP | Barros-Battesti and Arzua, 1997; Linardi and Guimarães, 2000; Horta et al., 2007; Muller et al., 2009; Oliveira et al., 2010; Brum, 2018 |
| *P. (N.) dentei* Guimarães, 1947 | **Rodentia:** *Akodon* *cursor*, *Akodon* *montensis*, *Delomys* *dorsalis*, *Oxymycterus* *quaestor* Thomas, 1903, *Thaptomys* *nigrita* | SP, RJ | Linardi and Guimarães, 2000; De Moraes et al., 2003; Linardi, 2011 |
| *P. (N.) frustratus* Johnson, 1957 | **Didelphimorphia:** *Didelphis* *marsupialis*, *Philander* *opossum*.  **Rodentia:** *Akodon* *montensis*, *Cavia* *aperea*, *Delomys* *dorsalis*, *Oxymycterus* *dasytrichus*, *Oxymycterus* *quaestor* Thomas, 1903, *Thaptomys* *nigrita*. | SP, RJ, SC, PR | Linardi and Guimarães, 2000; de Moraes et al., 2003; Linardi, 2011 |
| *P. (N.) pradoi* (Wagner, 1937) | **Carnivora:** *Nasua* *nasua*.  **Didelphimorphia:** *Didelphis* *albiventris*, *Didelphis* *marsupialis*, *Philander* *opossum*.  **Rodentia:** *Akodon* *cursor*, *Akodon* *montensis*, *Akodon* *reigi* González, Langguth & Oliveira, 1998, *Akodon* *serrensis*, *Euryoryzomys* *russatus*, *Euryzygomatomys* *spinosus*, *Necromys* *lasiurus*, *Nectomys* *squamipes*, *Oligoryzomys* *nigripes*, *Oxymycterus* *quaestor*, *Rattus* *rattus* *rattus*, *Thaptomys* *nigrita*, *Trinomys* *iheringi* (Thomas, 1911). | BA, ES, PR, RJ, RS, SC, SP | Barros et al., 1993; Hastriter and Peterson, 1997; Linardi and Guimarães, 2000; Carvalho et al., 2001; Linardi, 2011; Schott et al., 2020 |
| *P. (N.) pygaerus* (Wagner, 1937) | **Didelphimorphia:** *Didelphis* *aurita*  **Rodentia:** *Akodon* *cursor*, *Akodon* *montensis*, *Akodon* *serrensis*, *Euryoryzomys* *russatus*, *Necromys* *lasiurus*, *Nectomys* *squamipes*, *Oxymycterus* *quaestor*, *Rattus* *rattus* *rattus*, *Thaptomys* *nigrita*. | MG, PR, RJ, SC | Linardi and Guimarães, 2000; Linardi et al., 1987; Carvalho et al., 2001 |
| **Subgenus *Polygenis* (*Polygenis*) Jordan, 1939** |  |  |  |
| *P. (P.) acodontis* (Jordan & Rothschild, 1923) | **Rodentia:** *Guerlinguetus aestuans*. | SC | Linardi and Guimarães, 2000 |
| *P. (P.) adelus* (Jordan & Rothschild, 1923*)* | **Didelphimorphia:** *Monodelphis domestica*.  **Rodentia:** *Akodon montensis*, *Calomys tener* (Winge, 1887), *Cerradomys subflavus*, *Euryoryzomys russatus*, *Necromys lasiurus*, *Rhipidomys mastacalis*, *Trinomys albispinus*, *Trinomys setosus*, *Wiedomys pyrrhorhinos*. | BA, MG, PE, SP | Linardi and Guimarães, 2000; Linardi, 2011 |
| *P. (P.) axius axius* (Jordan & Rothschild, 1923) | **Didelphimorphia:** *Didelphis* *albiventris*, *Lutreolina* *crassicaudata*.  **Rodentia:** *Akodon* *cursor*, *Necromys* *lasiurus*, *Nectomys* *squamipes*, *Oxymycterus* *dasytrichus*. | MG, PR, RS, SP | Barros-Battesti and Arzua, 1997; Linardi and Guimarães, 2000; Linardi, 2011 |
| *P. (P.) axius pessoai* Guimarães, 1956 | **Rodentia:** *Cerradomys* *subflavus*, *Oligoryzomys* *nigripes*, *Oxymycterus* *dasytrichus*. | AL, PE | Linardi and Guimarães, 2000 |
| *P. (P.) axius proxima* Guimarães, 1948 | **Didelphimorphia:** *Lutreolina crassicaudata.*  **Rodentia:** *Akodon montensis, Necromys lasiurus*. | MG, MS, RS, SP | Linardi and Guimarães, 2000; Muller et al., 2009; Linardi, 2011; 2017 |
| *P. (P.) bohlsi bohlsi* (Wagner, 1901) | **Didelphimorphia:** *Didelphis* sp. Linnaeus, 1758.  **Rodentia:** *Calomys* *callosus* (Rengger, 1830), *Calomys* *tener*, *Cerradomys* *subflavus*, *Cuniculus* *paca*, *Necromys* *lasiurus*, *Nectomys* *squamipes*, *Oligoryzomys* *nigripes*, *Oxymycterus* *dasytrichus*, *Rattus* *rattus* *frugivorus*, *Thrichomys* *apereoides*. | ES, GO, MG, MS | Linardi and Guimarães, 2000; Pinto et al., 2009; de Sousa et al., 2017; 2018 |
| *P. (P.) bohlsi jordani* (Lima, 1937) | **Carnivora:** *Cerdocyon* *thous*, *Galictis* *vittata*.  **Didelphimorphia:** *Didelphis* *albiventris*, *Metachirus* *nudicaudatus**, *Monodelphis* *domestica*.  **Lagomorpha:** *Sylvilagus* *brasiliensis*.  **Primata:** *Callithrix* *jacchus* (Linnaeus, 1758).  **Rodentia:** *Akodon* *montensis*, *Calomys* *expulsus* (Lund, 1840), *Calomys* *tener*, *Cavia* *aperea*, *Cerradomys* *subflavus*, *Echimys* *chrysurus* (Zimmermann, 1780), *Euryoryzomys* *lamia* (Thomas, 1901), *Euryoryzomys russatus**, *Galea* *spixii*, *Holochilus* *brasiliensis*, *Holochilus* *sciureus*, *Kerodon* *rupestris*, *Mus* *musculus* *brevirostris*, *Necromys* *lasiurus*, *Nectomys* *squamipes*, *Oligoryzomys* *flavescens*, *Oligoryzomys* sp.*, *Oxymycterus* sp.*, *Rattus* *norvegicus*, *Rattus* *rattus* *alexandrinus*, *Rattus* *rattus* *frugivorus*, *Rhipidomys* *mastacalis*, *Thrichomys* *inermis*, *Thrichomys* *laurentius*, *Trinomys* *albispinus*, *Trinomys* *setosus*, *Wiedomys* *pyrrhorhinos*. | AL, BA, CE, PE, PB, RN, SP* | Almeida et al., 1986; Hastriter and Peterson, 1997; Linardi and Guimarães, 2000; Oliveira et al., 2009; de Oliveira et al., 2021; **this study*** |
| *P. (P.) occidentalis occidentalis* (Jordan & Rothschild, 1923) | **Carnivora:** *Cerdocyon* *thous*.  **Cingulata:** *Dasypus* *novemcinctus*.  **Didelphimorphia:** *Didelphis* *aurita*, *Didelphis* *marsupialis*.  **Rodentia:** *Akodon* spp. Meyen, 1833, *Delomys* *dorsalis*, *Guerlinguetus* *aestuans*, *Guerlinguetus* *brasiliensis* *ingrami* (Thomas, 1901), *Necromys* *lasiurus*, *Oligoryzomys* *nigripes*, *Oxymycterus* *nasutus* (Waterhouse, 1837), *Rattus* *norvegicus*, *Rhipidomys* *mastacalis*, *Scapteromys* *tumidus* (Waterhouse, 1837), *Thrichomys* *inermis*.  **Tinamiformes:** *Crypturellus* *obsoletus* *obsoletus* (Sclater, 1865). | AL, CE, ES, PR, RJ, RS, SC, SP | Linardi and Guimarães, 2000; Pinto et al., 2009; Oliveira et al., 2010; Linardi, 2011; Schott et al., 2020 |
| *P. (P.) occidentalis steganus* (Jordan & Rothschild, 1923) | **Rodentia:** *Rhipidomys* *mastacalis*. | CE, GO, PA, RR | Linardi and Guimarães, 2000 |
| *P. (P.) platensis platensis* (Jordan & Rothschild, 1908) | **Rodentia:** *Akodon azarae* (Fischer, 1829), *Ctenomys* *flamarioni* Travi, 1981, *Ctenomys* *minutus* Nehring, 1887, *Delomys* *dorsalis*, *Oligoryzomys* *nigripes*, *Scapteromys* *tumidus*. | RS | Linardi and Guimarães, 2000; Linardi et al., 2005; Schott et al., 2020 |
| *P. (P.) rimatus* (Jordan, 1932) | **Didelphimorphia:** *Didelphis* *albiventris*, *Didelphis* *marsupialis*, *Monodelphis* *brevicaudata*, *Philander* sp. Brisson, 1762, *Metachirus nudicaudatus**.  **Rodentia:** *Akodon* sp.*, *Akodon* *cursor*, *Akodon* *montensis*, *Akodon* *serrensis*, *Calomys* *expulsus*, *Cerradomys* *subflavus*, *Delomys* *dorsalis*, *Euryoryzomys* *russatus**, *Euryzygomatomys* *spinosus*, *Guerlinguetus* *brasiliensis* *ingrami*, *Necromys* *lasiurus*, *Nectomys* *squamipes*, *Oligoryzomys* *mattogrossae* Allen, 1916, *Oligoryzomys* *nigripes*, *Oxymycterus* sp.*, *Oxymycterus* *quaestor*, *Rattus* *norvegicus*, *Rattus* *rattus* *alexandrinus*, *Sooretamys* *angouya*, *Thaptomys* *nigrita*, *Trinomys* *dimidiatus*. | BA, ES, GO, MG, PA, PR, RJ, RS, SC, SP | Barros et al., 1993; Barros-Battesti and Arzua, 1997; Hastriter and Peterson, 1997; Linardi and Guimarães, 2000; Carvalho et al., 2001; Horta et al., 2007; Muller et al., 2009; Oliveira et al., 2010; Brum, 2018; Schott et al., 2020; **this study*** |
| *P. (P.) roberti beebei* (Fox, 1947) | **Rodentia:** *Euryoryzomys* spp. | AP | Linardi and Guimarães, 2000 |
| *P. (P.) roberti roberti* (Rothschild, 1905) | **Carnivora:** *Leopardus* *pardalis*.  **Chiroptera:** *Chrotopterus auritus* (Peters, 1856)*  **Cingulata:** *Dasypus* *novemcinctus*.  **Didelphimorphia:** *Didelphis* *albiventris*, *Didelphis* *aurita**, *Didelphis* *marsupialis*, *Gracilinanus* sp. Gardner & Creighton, 1989*, *Marmosa* (*Micoureus*) *paraguayanus*, *Metachirus* *nudicaudatus**, *Monodelphis* sp.*  **Pilosa:** *Tamandua* *tetradactyla*.  **Rodentia:** *Akodon* sp.*, *Akodon* *montensis*, *Brucepattersonius* sp. Hershkovitz, 1998*, *Cerradomys* *subflavus*, *Dasyprocta* *azarae* Lichtenstein, 1823, *Dasyprocta* (Linnaeus, 1758), *Delomys* *dorsalis*, *Euryoryzomys* *lamia*, *Euryoryzomys russatus**, *Guerlinguetus* *brasiliensis* *ingrami**, *Holochilus* *brasiliensis**, *Hylaeamys* *megacephalus* (Fischer, 1814)*, *Hylaeamys* *oniscus* (Thomas, 1904), *Nectomys* *squamipes**, *Oligoryzomys* *nigripes**, *Oxymycterus* sp.*, *Oxymycterus* *quaestor*, *Phyllomys* sp. Lund, 1839*, *Proechimys* *guyannensis* (Geoffroy, 1803), *Rattus* *norvegicus, Rhipidomys* *mastacalis**, *Sooretamys angouya**, *Thaptomys* *nigrita*, *Trinomys* *dimidiatus*, *Trinomys* *setosus*. | BA, ES, GO, MG, MS, PE, PR, RJ, RS, SC, SP | Barros et al., 1993; Hastriter and Peterson, 1997; Carvalho et al., 2001; Horta et al., 2007; Muller et al., 2009; Brum, 2018; Schott et al., 2020; **this study*** |
| *P. (P.) tripopsis* Guimarães, 1948 | **Carnivora:** *Leopardus* *pardalis*.  **Cingulata:** *Dasypus* *novemcinctus*.  **Rodentia:** *Cerradomys* *subflavus*, *Echimys* *chrysurus*, *Euryoryzomys* *lamia*, *Holochilus* *brasiliensis*, *Hylaeamys* *megacephalus*, *Necromys* *lasiurus*, *Oligoryzomys* *nigripes*, *Oxymycterus* *dasytrichus*, *Rhipidomys* *mastacalis*. | BA, CE, GO, MS, PE | Linardi and Guimarães, 2000; Sponchiado et al., 2015; Linardi, 2017 |
| *P. (P.) tripus* (Jordan, 1933) | **Didelphimorphia:** *Didelphis* *albiventris*, *Didelphis* *aurita*, *Didelphis* *marsupialis*, *Lutreolina* *crassicaudata, Monodelphis* *domestica*.  **Rodentia:** *Akodon* *cursor*, *Akodon* *montensis*, *Calomys* *expulsus*, *Calomys* *tener*, *Cavia* *aperea*, *Cerradomys* *subflavus*, *Euryzygomatomys* *spinosus*, Galea *spixii*, *Holochilus* *brasiliensis*, *Holochilus* *sciureus,* *Mus* *musculus* *brevirostris*, *Necromys* *lasiurus*, *Nectomys* *squamipes*, *Oligoryzomys* *nigripes*, *Oxymycterus* *dasytrichus*, *Rattus* *norvegicus*, *Rattus* *rattus* *alexandrinus*, *Rattus* *rattus* *frugivorus*, *Rhipidomys* *mastacalis*, *Thrichomys* *apereoides*, *Thrichomys* *inermis*, *Thrichomys* *laurentius*, *Trinomys* *albispinus*, *Trinomys* *setosus*, *Wiedomys* *pyrrhorhinos*. | AL, BA, CE, ES, GO, MG, PE, PR, RJ, RN, SP | Botelho et al.,1981; Almeida et al., 1986; Botelho; Linardi, 1992; Barros-Battesti and Arzua, 1997; Hastriter and Peterson, 1997; Linardi and Guimarães, 2000; Carvalho et al., 2001; Horta et al., 2007; Oliveira et al., 2009; Pinto et al., 2009 |
| **Tribe Rhopalopsyllini Oudemans, 1909** |  |  |  |
| **Genus *Gephyropsylla Barrera, 1952*** |  |  |  |
| *G. klagesi klagesi* (Rothschild, 1904) | **Cingulata:** *Dasypus* *novemcinctus*.  **Didelphimorphia:** *Didelphis* spp., *Philander* *opossum*.  **Rodentia:** *Cerradomys* *subflavus*, *Proechimys* *guyannensis*, *Rhipidomys* *mastacalis*. | AM, CE, GO, PA, RR | Rafael, 1982; Linardi et al., 1991; Linardi and Guimarães, 2000 |
| *G. klagesi samuelis* (Jordan & Rothschild, 1923) | **Didelphimorphia:** *Didelphis* *marsupialis*.  **Rodentia:** *Holochilus* *brasiliensis*, *Proechimys* *guyannensis*, *Proechimys* *longicaudatus* (Rengger, 1830). | AM, GO, RO, RR | Linardi et al., 1991; Hastriter and Peterson, 1997; Linardi and Guimarães, 2000 |
| **Genus *Hechtiella* Barrera, 1952** |  |  |  |
| *H. lakoi* (Guimarães, 1948) | **Didelphimorphia:** *Philander* *opossum*.  **Rodentia:** *Euryoryzomys* *lamia*, *Oligoryzomys* *nigripes*, *Philander*, *Trinomys* *dimidiatus*, *Trinomys* *iheringi*. | ES, MG, RJ, SP | Linardi and Guimarães, 2000; Bittencourt and Rocha, 2003; de Moraes et al., 2003; Linardi, 2011 |
| *H. lopesi* Guimarães & Linardi, 1993 | **Rodentia:** *Proechymis* spp., *Trinomys iheringi*. | SP | Guimarães and Linardi, 1993, Linardi and Guimarães, 2000; Linardi, 2011 |
| *H. nitidus* (Johnson, 1957) | **Cingulata:** *Dasypus* *novemcinctus.*  **Didelphimorphia:** *Didelphis* *marsupialis*, *Marmosops* *incanus*, *Metachirus* *nudicaudatus.*  **Rodentia:** *Necromys* *lasiurus*, *Nectomys* *squamipes*, *Trinomys* *dimidiatus*, *Trinomys* *iheringi*, *Trinomys* *paratus*. | BA, ES, MG, RJ | Botelho et al.,1981; Hastriter and Peterson, 1997; Linardi and Guimarães, 2000; Pinto et al., 2009 |
| **Genus *Rhopalopsyllus* Baker, 1905** |  |  |  |
| *R. australis australis* Rothschild, 1904 | **Perissodactyla:** *Tapirus terrestris*.  **Rodentia:** *Dasyprocta* *fuliginosa* Wagler, 1832, *Proechimys* *guyannensis*. | AP, PA, RO, RR | Linardi et al., 1991; Linardi and Guimarães, 2000; Gonçalves et al; 2023 |
| *R. australis tamoyus* Jordan & Rothschild, 1923 | **Artiodactyla:** *Mazama* *rufa* Illiger, 1815.  **Carnivora:** *Eira* *barbara*, *Nasua* *nasua*, *Procyon* *cancrivorus*.  **Cingulata:** *Dasypus* *novemcinctus*.  **Pilosa:** *Tamandua* spp. Gray, 1825.  **Rodentia:** *Cuniculus* *paca*, *Dasyprocta* *azarae*, *Dasyprocta* *fuliginosa*. | GO, MG, MS, MT, RO, SP | Linardi and Guimarães, 2000; Linardi, 2011; 2017; Mendonça et al., 2020 |
| *R. australis tupiniquinus* Guimarães, 1940 | **Carnivora:** *Eira* *barbara*, *Leopardus* *pardalis*. | SP | Linardi and Guimarães, 2000; Linardi, 2011 |
| *R. australis tupinus* Jordan & Rothschild, 1923 | **Rodentia:** *Myoprocta acouchy* (Erxleben, 1777). | PA | Linardi and Guimarães, 2000 |
| *R. crypturi* Wagner, 1939 | **Tinamiformes:** *Crypturellus obsoletus obsoletus.* | SC | Linardi and Guimarães, 2000 |
| *R. garbei Guimarães, 1940* | **Rodentia:** *Myoprocta acouchy.* | PA | Linardi and Guimarães, 2000 |
| *R. lugubris lugubris* Jordan & Rothschild, 1908 | **Artiodactyla:** *Mazama* *americana* (Erxleben, 1777).  **Cingulata:** *Dasypus* *novemcinctus.*  **Didelphimorphia:** *Didelphis* *marsupialis.*  **Rodentia:** *Akodon* *montensis*, *Cuniculus* *paca*, *Dasyprocta* *leporina*, *Oxymycterus* *quaestor*, *Trinomys* *dimidiatus*, *Trinomys* *iheringi*. | ES, GO, MG, MS, MT, PA, RJ, RO, SC, SP | Linardi and Guimarães, 2000; Horta et al., 2007; Silveira, 2012; Linardi, 2017, Mendonça et al., 2020 |
| *R. lutzi lutzi* (Baker, 1904) | **Carnivora:** *Canis lupus familiaris*, *Cerdocyon* *thous*, *Leopardus* *pardalis*, *Nasua* *nasua*, *Puma* *yagouaroundi*, *Galictis* *vittata*.  **Cingulata:** *Dasypus* *novemcinctus.*  **Didelphimorphia:** *Didelphis* *albiventris*, *Didelphis* *aurita*, *Didelphis* *marsupialis*, *Philander* *opossum*.  **Pilosa:** *Tamandua* *tetradactyla*.  **Rodentia:** *Akodon* *serrensis*, *Dasyprocta* *azarae*, *Dasyprocta* *leporina*. | BA, ES, GO, MG, MS, PR, RJ, RO, SP | Barros et al., 1993; Barros-Battesti and Arzua; 1997 Cerqueira et al., 2000; Linardi and Guimarães, 2000; Scofield et al., 2005; Rodrigues et al., 2006; Horta et al., 2007; Rodrigues et al., 2008; Pinto et al., 2009; Linardi, 2017; Estevam et al., 2020 |
| *R. saevus* Jordan & Rothschild, 1923 | **Cingulata:** *Dasypus* *novemcinctus.*  **Didelphimorphia:** *Didelphis* *marsupialis.*  **Rodentia:** *Dasyprocta fuliginosa*. | MT, RO | Linardi; Guimarães, 2000, Mendonça et al., 2020 |
| **Family Stephanocircidae Wagner, 1928** |  |  |  |
| **Subfamily Craneopsyllinae Wagner, 1939** |  |  |  |
| **Tribe Craneopsyllini Wagner, 1939** |  |  |  |
| **Genus *Craneopsylla* Rothschild, 1911** |  |  |  |
| *C. minerva minerva* (Rothschild, 1903) | **Chiroptera:** *Anoura* *geoffroyi* *geoffroyi* Gray, 1838, *Sturnira* (*Sturnira*) *lilium* (Geoffroy, 1810).  **Didelphimorphia:** *Didelphis* *albiventris*, *Lutreolina* *crassicaudata*, *Marmosops* *incanus*, *Monodelphis* *domestica*, *Philander* *opossum*.  **Rodentia:** *Akodon* *cursor*, *Akodon* *montensis*, *Akodon reigi*, *Akodon* *serrensis*, *Brucepattersonius iheringi*, *Calomys* *tener*, *Cerradomys* *subflavus*, *Delomys* *dorsalis*, *Euryoryzomys* *lamia*, *Euryoryzomys* *russatus*, *Guerlinguetus* *aestuans*, *Holochilus* *sciureus*, *Necromys* *lasiurus*, *Nectomys* *squamipes*, *Oligoryzomys* *flavescens*, *Oligoryzomys* *nigripes*, *Oxymycterus* *dasytrichus*, *Oxymycterus* *quaestor*, *Proechimys* *guyannensis*, *Rattus rattus rattus*, *Rhipidomys* *mastacalis*, *Sooretamys* *angouya*, *Thaptomys* *nigrita*, *Trinomys* *dimidiatus*, *Wiedomys* *pyrrhorhinos*. | AL, BA, CE, MG, PE, PR, RJ, RS, SC, SP | Linardi; Guimarães, 2000; Carvalho et al., 2001; Bittencourt and Rocha, 2003; Schott et al., 2020 |
| **Family Tungidae Taschenberg, 1880** |  |  |  |
| **Subfamily Hectopsyllinae Baker, 1904** |  |  |  |
| **Genus *Hectopsylla* Frauenfeld, 1860** |  |  |  |
| *H. psittaci* Frauenfeld, 1860 | **Columbiformes:** *Columba* *livia* Gmelin, 1789.  **Passeriformes:** *Progne* *chalybea* Gmelin, 1789, *Turdus* *leucomelas* Vieillot, 1818. | RJ, RS, SP | Linardi and Guimarães, 2000 |
| *Hectopsylla pulex* (Haller, 1880) | **Chiroptera:** *Histiotus* *velatus* (Geoffroy, 1824), *Molossus* *molossus* Pallas, 1766, *Molossus* *rufus* Geoffroy, 1805, *Peropteryx* *macrotis* (Wagner, 1843), *Phyllostomus* *hastatus*. | BA, MG, PE, PR, RJ, RS, SC, SP | Linardi and Guimarães, 2000; Esbérard, 2001; Luz et al., 2009 |
| **Subfamily Tunginae Taschenberg, 1880** |  |  |  |
| **Genus *Tunga* Jarocki, 1838** |  |  |  |
| *T. bondari* Wagner, 1932 | **Cariamiformes:** *Cariama cristata* (Temminck, 1823).  **Pilosa:** *Tamandua tetradactyla.* | BA, MG, SP | Hopkins and Rothschild, 1956; Linardi and Guimarães, 2000 |
| *T. bossii* Avelar, Linhares & Linardi, 2012 | **Rodentia:** *Delomys dorsalis.* | RJ | Linardi and Guimarães, 2000; [de Avelar et al., 2012](https://link.springer.com/article/10.1007/s00436-014-4081-8#ref-CR23) |
| *T. caecata* (Enderlein, 1901) | **Rodentia:** *Mus musculus, Rattus rattus rattus, Rattus norvegicus, Akodon cursor, Necromys pixuna, Nectomys squamipes, Oligoryzomys nigripes, Oxymycterus sp., Rhypidomys mastacalis.* | MG, PR, SP, RJ | Linardi and Guimarães, 2000; Linardi and Avelar, 2014 |
| *T. hexalobulata* Avelar, Facury Filho & Linardi, 2013 | **Artiodactyla:** *Bos taurus indicus.* | MG | [de Avelar et al., 2013](https://link.springer.com/article/10.1007/s00436-014-4081-8#ref-CR24) |
| *T. penetrans* (Linnaeus, 1758) | **Artiodactyla:** *Bos taurus indicus*, *Sus scrofa* Linnaeus*,* 1758, *Capra* *hircus* Linnaeus*,* 1758, *Ovis* *aries* Linnaeus*,* 1758, *Pecari* *tajacu* Linnaeus*,* 1758.  **Carnivora:** *Canis lupus familiaris, Felis cattus* Linnaeus*,* 1758*, Panthera onca*.  **Cingulata:** *Dasypus novemcinctus*.  **Passeriformes:** *Volatinia jacarina* (Linnaeus, 1766).  **Perissodactyla:** *Equus caballus* Linnaeus*,* 1758, *Tapirus terrestris*.  **Pilosa:** *Tamandua tetradactyla, Myrmecophaga tridactyla* (Linnaeus, 1758).  **Primata**: *Alouatta guariba clamitans* Cabrera, 1940, *Homo sapiens*.  **Rodentia:** *Cuniculus paca, Mus musculus*, *Rattus rattus rattus, Rattus norvegicus.* | AC, AL, AM, AP, BA, CE, DF, ES, GO, MA, MG, MS, MT, PA, PB, PE, PI, PR, RJ, RN, RO, RS, RR, SC, SE, SP, TO | Linardi, 1998; Linardi and Guimarães, 2000; Frank et al., 2012; de Avelar et al., 2013; Linardi and de Avelar, 2014; Schott et al., 2020; Tancredi et al., 2021; Santos et al., 2022; Jesus et al., 2023 |
| *T. terasma* Jordan, 1937 | **Cingulata:** *Cabassous unicinctus* (Linnaeus, 1758), *Dasypus novemcinctus, Euphractus sexcinctus* (Linnaeus, 1758), *Priodontes maximus* (Kerr, 1792). | ES, GO, MA, MG, MS, SP | Linardi and Guimarães, 2000; Antunes et al., 2006; Linardi and de Avelar, 2014 |
| *T. travassosi* Pinto & Dreyfus, 1927 | **Cingulata:** *Dasypus novemcinctus*. | MG, SP | Pinto and Dreyfus; 1927; Linardi and Guimarães, 2000; Linardi and de Avelar, 2014 |
| *T. trimamillata Pampiglione,* Trentini, Fioravanti, Onore & Rivasi, 2002 | **Artiodactyla:** *Bos* *taurus indicus, Sus scrofa, Capra* *hircus*, *Ovis* *aries.*  **Primata:** *Homo sapiens.*  **Rodentia:** *Hydrochoerus hydrochaeris*. | MG, SP | Fioravanti et al., 2003; Linardi and de Avelar, 2014; Harvey et al., 2021 |

Legend: *new hosts or locations provided in this study, AC: Acre, AL: Alagoas, AM: Amazonas, AP: Amapá, BA: Bahia, CE: Ceará, DF: Distrito Federal, ES: Espírito Santo, GO: Goiás, MA: Maranhão, MG: Minas Gerais, MS: Mato Grosso do Sul, MT: Mato Grosso, PA: Pará, PB: Paraíba, PE: Pernambuco, PI: Piauí, PR: Paraná, RJ: Rio de Janeiro, RN: Rio Grande do Norte, RO: Rondônia, RS: Rio Grande do Sul, RR: Roraima, SC: Santa Catarina, SE: Sergipe, SP: São Paulo, TO: Tocantins.
